# ToolConnect: A Functional Connectivity Toolbox for *In vitro* Networks

**DOI:** 10.3389/fninf.2016.00013

**Published:** 2016-03-30

**Authors:** Vito Paolo Pastore, Daniele Poli, Aleksandar Godjoski, Sergio Martinoia, Paolo Massobrio

**Affiliations:** ^1^Neuroengineering and Bio-Nano Technology Lab, Department of Informatics, Bioengineering, Robotics, System Engineering, University of GenoaGenoa, Italy; ^2^Institute of Biophysics, National Research CouncilGenova, Italy

**Keywords:** functional connectivity, *in vitro*, micro-electrode arrays, multi-threading, windows form application, neural networks, correlation algorithms, information theory algorithms

## Abstract

Nowadays, the use of *in vitro* reduced models of neuronal networks to investigate the interplay between structural-functional connectivity and the emerging collective dynamics is a widely accepted approach. In this respect, a relevant advance for this kind of studies has been given by the recent introduction of high-density large-scale Micro-Electrode Arrays (MEAs) which have favored the mapping of functional connections and the recordings of the neuronal electrical activity. Although, several toolboxes have been implemented to characterize network dynamics and derive functional links, no specifically dedicated software for the management of huge amount of data and direct estimation of functional connectivity maps has been developed. toolconnect offers the implementation of up to date algorithms and a user-friendly Graphical User Interface (GUI) to analyze recorded data from large scale networks. It has been specifically conceived as a computationally efficient open-source software tailored to infer functional connectivity by analyzing the spike trains acquired from *in vitro* networks coupled to MEAs. In the current version, toolconnect implements correlation- (cross-correlation, partial-correlation) and information theory (joint entropy, transfer entropy) based core algorithms, as well as useful and practical add-ons to visualize functional connectivity graphs and extract some topological features. In this work, we present the software, its main features and capabilities together with some demonstrative applications on hippocampal recordings.

## Introduction

In the last years, one of the major issues of computational neuroscience has been to understand the organization principles that rule brain connectivity datasets. Nowadays, it is widely accepted that simple motor behaviors and complex cognition tasks arise from the interactions of neuronal assemblies (Sporns, 2013). In addition, thanks to the development of new non-invasive approaches, a large amount of information regarding the structural organization and functional association of different brain areas have been obtained (Bandettini, 2012). Accurate and detailed studies for reconstructing anatomical connectivity have been performed, and a description of the brain's *structural connectivity* (i.e., the connectome) is partly available (Sporns et al., 2005). Indeed, structural connectivity drives and influences the network dynamics expressed by a neuronal system: from a connectivity point of view, the possible “modes of activity” define the *functional connectivity* of the same system (Feldt et al., 2011). Functional connectivity refers to the magnitude of temporal correlation in activity occurring between different neurons (or different neuronal assemblies) without any underlying causal model. The characterization of functional networks can be performed by using different families of 2-D and 1-D signals, such as neuro-images and electrophysiological recordings. Concerning *in vivo* data captured from different acquisition systems like EEG, ECoG, MEG, fMRI, a plethora of software has been developed to infer functional networks: Eeglab (Delorme and Makeig, 2004), eConnectome (He et al., 2011), NIPy (Millman and Brett, 2007), Trentool (Lindner et al., 2011) are some available custom software solutions developed to process the recorded data operating in a user friendly interface. In addition, an important contribution to the characterization of the estimated network maps comes from the Brain Connectivity Toolbox devised by the group of Sporns (Rubinov and Sporns, 2010) which embodies a rich collection of metrics developed in different programming languages (Matlab, Python, and C++) used to characterize the graphs describing the connectivity of neuronal networks.

The aim of this work is to present a novel toolbox which collects several statistical methods suitable to infer functional connectivity in neuronal assemblies. In the panorama of the already available tools, our software, named ToolConnect, has been customized to be applied to a peculiar kind of neuronal preparation: *in vitro* dissociated neuronal networks coupled to Micro-Electrode Arrays (MEAs). The possibility to use *in vitro* reduced models of neuronal assemblies coupled to MEAs to investigate electrophysiological principles of complex brain networks is attracting an increasing number of scientists. By exploiting a reduced degree of complexity and consequently a better observability and controllability of some variables than in the intact brain, several investigations were performed. Starting from the pioneering works of Gross at the beginnings of the nineties (Gross et al., 1992) where neuronal networks were used for biochemical sensing, this experimental model has been used to investigate different topics such as network development (Wagenaar et al., 2006), synaptic plasticity (Marom and Shahaf, 2002; Chiappalone et al., 2008), response to electrical stimulation (Wagenaar et al., 2004; Le Feber et al., 2010), presence of self-organized critical states (Pasquale et al., 2008; Massobrio et al., 2015), generation of peculiar patterns of activity (Rolston et al., 2007), neurotoxicity (Defranchi et al., 2011) up to closed-loop experiments (Wagenaar et al., 2005b; Bonifazi et al., 2013).

During these years, the dynamics of *in vitro* dissociated networks have been widely studied, and, consequently, some analysis tools have been already released: software like Mea Tools (Egert et al., 2002), MeaBench (Wagenaar et al., 2005a), Find (Meier et al., 2008), SpyCode (Bologna et al., 2010), or QSpike (Mahmud et al., 2014) collect several algorithms to perform spike and burst detections, as well as statistics like inter spike/burst interval distributions, firing/bursting rate, etc. However, to the best of our knowledge, there are still no available automated customized tools suitable for managing and inferring functional connectivity from spike trains acquired with MEAs. In order to provide such a computational tool in a flexible and friendly environment for the neuroscience researcher's community, we designed ToolConnect, an open source software which collects several algorithms we developed in our lab for inferring functional connectivity in dissociated neuronal networks. The current version implements correlation- (cross-correlation, partial-correlation) and information theory (joint entropy, transfer entropy) based core algorithms. ToolConnect has been implemented as a standalone windows GUI application, using C# programming language with Microsoft Visual Studio based on .NET framework 4.5 development environment. These tools provide the developer with a relatively simple and efficient memory management, thread-code execution, a simple events management and powerful instruments to build a friendly GUI. The toolbox consists of several modules in the windows form embodiment and allow the user to easily manipulate and analyze data, while providing computational efficiency and accuracy. ToolConnect produces both numerical and graphical results. Starting from the intrinsic output of the algorithms (e.g., correlation matrices), we implemented add-on functions based on the graph theory, that produce some relevant metrics (e.g., clustering coefficient, path length, degree distribution, etc.) commonly used to characterize the topological features of the obtained graphs.

In the next sections of this paper, we present the software architecture, the implemented algorithms and few examples of analysis performed on hippocampal neuronal networks recorded by conventional and high density MEAs.

## Materials and methods

In this Section, we present a brief survey of the statistical algorithms we implemented to derive the different functional connectivity maps, in order to provide the reader with a basic theoretical background. The last part of this section briefly describes the Receiver Operating Characteristic (ROC) curves we used to assess the accuracy performance of the implemented algorithms.

### Cross-correlation

Let *x* and *y* be two spike trains recorded from two electrodes; let *T* be a time frame around the spikes of the train *x* and Δτ the bin amplitude usually set at multiple of the sampling frequency.

The cross-correlation function *C*_*xy*_(τ) is defined as follows (Eytan et al., 2004; Garofalo et al., 2009):

(1) $$C_{xy}\left(\tau \right)=\frac{1}{\sqrt{N_{x}N_{y}}}\sum_{s=1}^{N_{x}}\overset{t_{i}=(\tau +\frac{\Delta \tau }{2})}{\underset{t_{i}=(\tau -\frac{\Delta \tau }{2})}{\sum }}x(t_{s})y(t_{s}-t_{i})$$

where *t*_*s*_ is the timing of a spike in the train *x*, while *N*_*x*_ and *N*_*y*_ are the total number of spikes in the trains *x* and *y* respectively. Equation (1) corresponds to a normalized form, in order to have *C*_*xy*_(τ) values belonging to the interval [0, 1]. The symmetry between *C*_*xy*_(τ) and *C*_*yx*_(τ) is maintained since: *C*_*xy*_(τ) = *C*_*yx*_(−τ).

### Partial-correlation

Cross-Correlation (CC) is not able, by definition, to distinguish between direct and indirect connections (Eichler et al., 2003). Partial coherence (Brillinger et al., 1976), instead, allows to assess the dependence between two spike trains, removing the effects of the activity of all other spike trains. Let us define *S*_*xy*_ as the cross spectrum between electrodes *x* and *y*, and *P* as the population of all the electrodes except *x* and *y*. The partialization procedure consists to subtract from *S*_*xy*_ the effects of all spike trains of the population *P* as follows:

(2) $$S_{xy|P}=S_{xy}-\left(S_{xP}\;\;S_{PP}{}^{-1}\;S_{yP}\right)$$

where *S*_*xP*_ is the cross spectrum of neuron *x* vs. the population *P, S*_*yP*_ is the cross spectrum of neuron *y* vs. the population *P*, and *S*_*PP*_ is the cross spectrum between all the neurons of the population without *x* and *y*. After the partialization, if two connections converge to the same node, the two input nodes can become correlated as an artifact. This intrinsic condition of the method is known as *marrying- parents effect* (Eichler et al., 2003).

### Transfer Entropy

Transfer Entropy (TE) is an information theory method that allows to extract the causal relationships from time series (Lungarella et al., 2007) and, differently from CC and PC, it is not symmetric. Let *x* and *y* be two spike trains, we define Transfer Entropy in the following way (Garofalo et al., 2009):

(3) $$TE_{y->x}=\sum_{x_{t},x_{t-1,}y_{t-1}}p\left(x_{t},x_{t-1},y_{t-1}\right)log\frac{p\left(x_{t}|x_{t-1},y_{t-1}\right)}{p\left(x_{t}|x_{t-1}\right)}$$

where *x*_*t*_ and *x*_*t*−1_ are the present and the past of *x*, respectively. Transfer Entropy is able to detect the information flow between two different nodes by estimating the part of a neuron activity which does not depend on its own past, but which depends on another neuron's past activity.

### Joint Entropy

Joint Entropy (JE) is an entropic measure of the cross inter spike intervals (cISIs) (Garofalo et al., 2009). Let us consider a reference spike train *x* and a target spike train *y*, divided into temporal bins of any size. Given a spike in the *x* at the instant *t*_*x*_, and supposing that the subsequent spike in *y* arises at the instant *t*_*y*_, the cISI is defined as the temporal difference between the aforementioned spikes:

(4) $$cISI=t_{y}-\;t_{x}$$

Mathematically we can define JE as:

(5) $$JE\;\left(x,y\right)=\;-\;\overset{n}{\underset{k=1}{\sum }}p(cISI_{k})\log_{2}p\left(cISI_{k}\right)$$

where *p*(*cISI*_*k*_) is the probability to encounter a cISI of size *k* bins. The higher are JE's values, the lower are the probabilities that the electrode correspondent to the train *y* is firing as a consequence of the one correspondent to *x*. Lower values of JE, instead, correspond to high probability to have a connection among the analyzed electrodes.

### Receiver Operating Characteristic (ROC) Curve

The ROC curve (Fawcett, 2006) is a well-known technique for evaluating the performances of binary classifiers. A ROC curve is obtained by comparing the Synaptic Weight Matrix (SWM) of a neural network and the estimated Thresholded Connectivity Matrix (TCM). For a given threshold, all the TCM elements are considered as possible functional connections. If a non-zero element of the TCM corresponds to an existing connection (a non-zero value in the SWM), it is considered as a True Positive (TP), while if it corresponds to a zero value in the SWM, then it is considered as a False Positive (FP). Furthermore, the TCM elements equal to zero either correspond to an existing connection (a non-zero value in the SWM), called False Negative (FN), or they correspond to a null element, called True Negative (TN). Sweeping the threshold from the 0.5 percentile to the 99.5 percentile, a variable number of TPs, FPs, TNs, and FNs are obtained. Finally, the ROC curve is built by reporting on a two-dimensional plot the true positive rate (TPR) and the false positive rate (FPR) defined as follows:

(6) $$TPR=\;\frac{TP}{TP+FN}$$

(7) $$FPR=\;\frac{FP}{FP+TN}$$

To reduce the ROC curves to a single scalar value it is possible to consider the Area Under Curve (AUC). A random guess corresponds to an AUC value of 0.5.

## Results

The use of MEAs to record electrophysiological signals coming from neuronal networks produce a huge amount of data depending of the number of recording sites and acquisition time. As an example, a recording of 10 min with a sampling frequency of 10 kHz produces 6·10^6^ samples per electrode. Recent technological efforts brought to MEAs acquisition system with thousands of electrodes. For instance, the *Active Pixel Sensor* (*APS*) array (Berdondini et al., 2009) and the *high-density CMOS* array (Matsuda et al., 2003) make use of 4096 and 11,000 electrodes respectively; using these recording systems means dealing with a huge amount of data. Consequently, a functional connectivity analysis requires a smart software, capable to provide an efficient custom memory management strategy, while being very simple in the usage. It is a common practice that a complete experimental dataset includes several experiments, each of one is split into several phases to be analyzed individually. For this reason, we implemented a multiple experiments analysis procedure, which automatically detects the experiments to be analyzed and performs the needed analysis on the different sets of data. Our approach to the multiple experiments analysis implementation relies upon a specific directory tree structure of the input files (i.e., the electrode's text file) that is the only pre-requisite for a correct analysis of the recorded data. First, it is necessary to choose the root folder which contains all the experiments' data. Then, an *ad-hoc* Graphical User Interface (GUI) allows the user to perform the functional connectivity analysis on the entire experimental set, or to manually select the experiments to analyze. The different experiments are analyzed sequentially with a progress bar indicating the percentage of progress for any of the implemented connectivity methods. To summarize, multiple analysis, optimization of the implemented algorithm (including a thresholding procedure), an intuitive GUI, and the minimization of the computational time are the main features of ToolConnect. In the next sections, we describe the software architecture and the specific implementation strategies.

### Software architecture

We implemented ToolConnect as a standalone windows GUI application, using C# programming language with Microsoft Visual Studio based on .NET framework 4.5 development environment. These tools provide the developer with a relatively simple and efficient memory management, thread-code execution, simple events management and powerful instruments to build a friendly GUI. We developed, tested and used ToolConnect on Microsoft Windows (versions 7 or higher).

**Figure 1:**
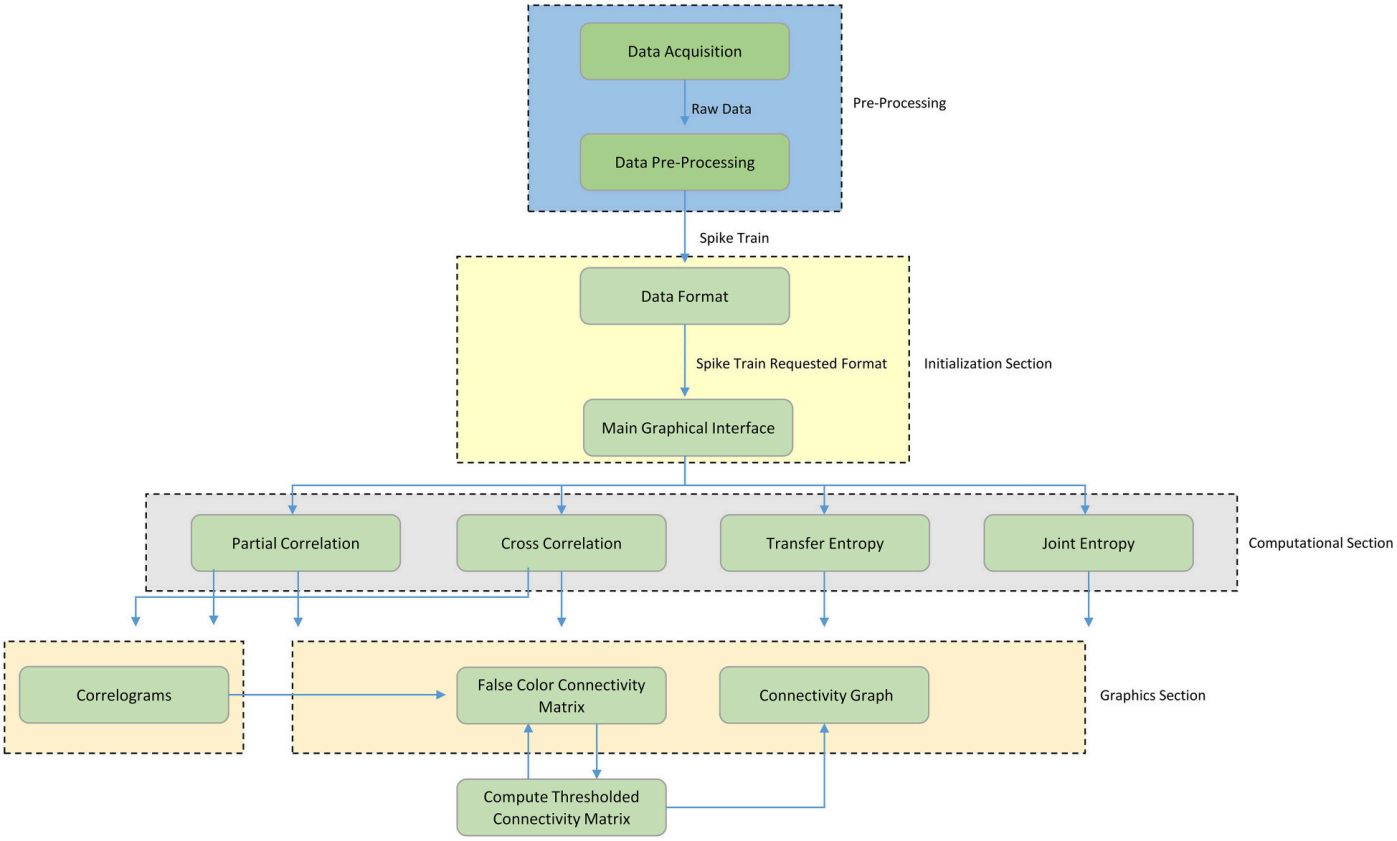
**Schematic overview of TOOLCONNECT**. The Functional blocks show the computational and graphical tools embedded in the software package. The flow chart starts with the pre-processing section; it includes the data acquisition procedure and all the other operations necessary to create the spike trains, which are the software's input data.

Figure 1 shows a schematic overview of the ToolConnect's functional blocks; the first blue dashed block represents the Pre-Processing section (not implemented in ToolConnect), needed to produce the input spike trains in an appropriate format for the further analysis. The current version of ToolConnect manages only point processes (e.g., spike trains) of specific data formats. The initialization section (yellow dashed block) describes the data formatting operations and the main graphical interface blocks. The former represents the necessary operations to convert the spike trains into the specific format to provide as input to the implemented methods; the latter describes the computational analysis that the user can perform, as well as the use of the graphical tools embedded in the program. The computational section (gray dashed block) includes the implemented methods that perform the functional connectivity analysis: Partial Correlation (PC), Cross-Correlation (CC), Transfer Entropy (TE), and Joint Entropy (JE). Finally, the graphical section (pink dashed block) includes the entire set of graphical tools embedded in the program: the plot of the correlograms for cross- and partial correlation, the computation, thresholding procedure and plotting of the Connectivity Matrix and the generation of the connectivity graph of the thresholded Connectivity Matrix. The following sections provides a detailed description of each block.

#### Initialization section

##### Data format

ToolConnect processes spike trains as input data; Input is formatted as text files (.txt file format), and the spike train relative to each channel needs to be stored in a single file. More specifically, each file contains the time stamps corresponding to the detected spikes. Thus, each file stores a sequence of integers, where the first element is the total number of sample in that particular experimental session, while the other elements represent the sample number correspondent to each spike (e.g., if an element of the sequence is equal to *n*, it means that a spike was recorded at the *nth* sample).

ToolConnect is independent from the acquisition system specifics or the MEA layout (i.e., number of microelectrodes of the array and spatial organization). Thus, no choices have been done *a priori* to adapt the file format to the peculiar acquisition systems. At present, we tested ToolConnect on spike trains recorded with the MEA60 and the MEA2100 acquisition systems of Multi Channel Systems (www.multichannelsystems.com; MCS, Reutlingen, Germany) and the BioCam acquisition system of 3Brain (www.3brain.com; Landquart, Switzerland).

##### Main graphical interface

**Figure 2:**
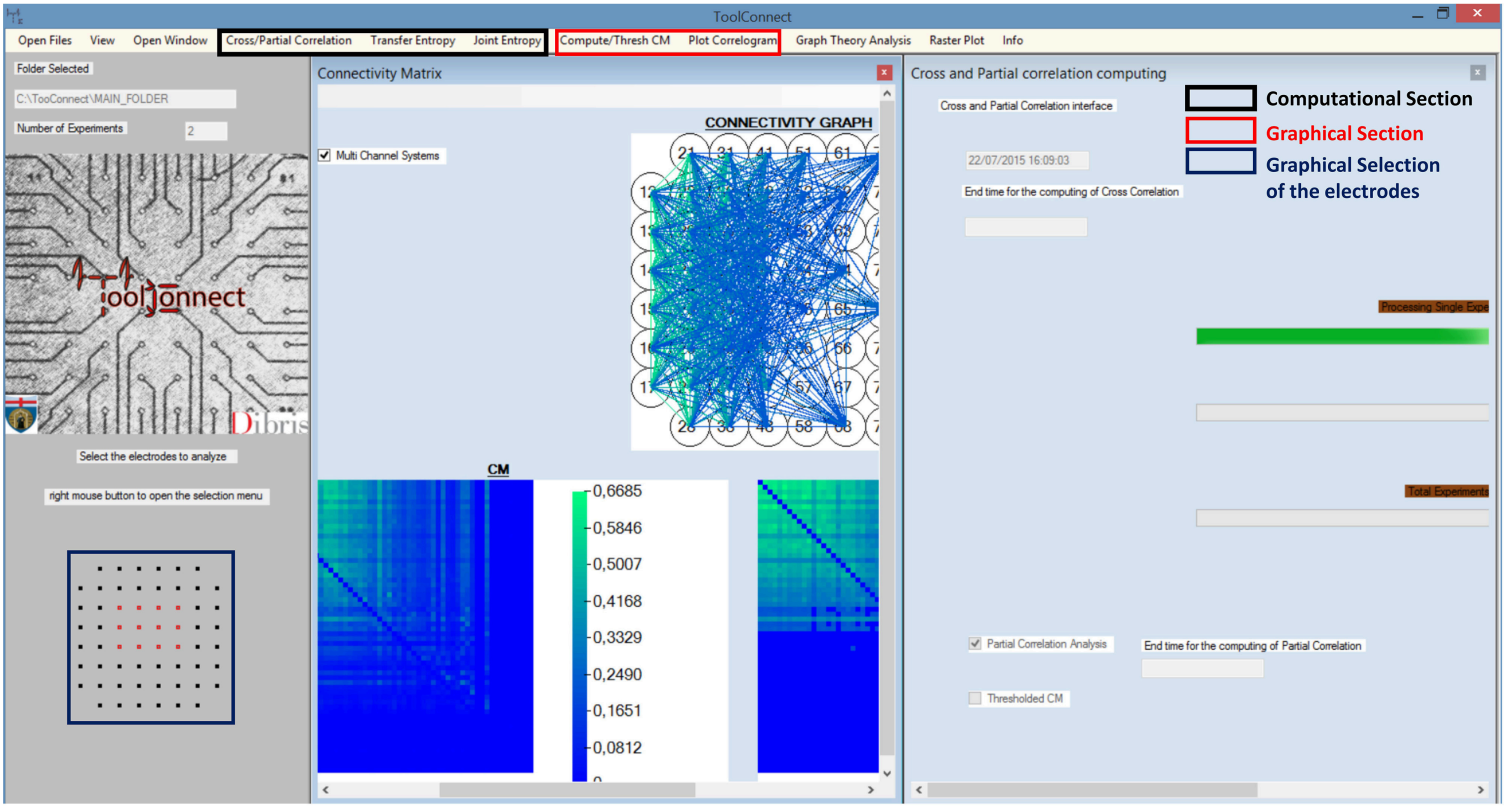
**toolconnect's GUI**. The main Graphic user interface is running under Windows; in the bottom left (blue box), it is possible to see the graphic Selection of the Electrodes to analyze (Electrodes format MEA 60 Multichannel System). The red box shows the components of the graphical section; the black box indicates the one relative to the computational section. In this example, the program was used to work with a CM (plot the CM, the TCM and the connectivity graph) and to perform a cross and partial correlation analysis.

We designed and developed ToolConnect taking care to satisfy the user-friendliness pre-requisite. Thus, our software package offers a GUI, which permits also inexperienced users to perform the needed functional connectivity analysis, to graphically represent the results, without knowing the details of the underlying algorithms and their implementation. Figure 2 shows a screenshot of ToolConnect's GUI. The GUI offers a drop-down menu that allows the selection and opening of the addressed interfaces designed for the Computational and the Graphical sections; these interfaces provide submenus, which make possible to set all the input parameters (more detail are reported in the Supplementary Materials).

Moreover, ToolConnect's GUI provides the user with the possibility to graphically select the electrodes to analyze (an example of this graphics selection is showed in Figure 2, bottom left, blue square). In the current version of the software, the subset of electrodes selection has been conceived for the main commercial acquisition systems, that are the 60, 120, and 252 MCS MEAs, the 64 electrodes Panasonic MED64 (Osaka, Japan), the 4096 electrodes 3Brain APS system. However, it is possible to extend these features to other acquisition platforms. If the acquisition system is not recognized, ToolConnect still allows the user to perform the functional connectivity analysis, automatically selecting the whole set of electrodes (all the text files in the data's input folder).

#### Implementation strategies section

In this Section, we describe the implementation and computational optimization strategies that we followed to develop the algorithms currently included in ToolConnect: cross-correlation, partial correlation, transfer entropy and joint entropy. All the algorithms share the same initialization procedure.

**Figure 3:**
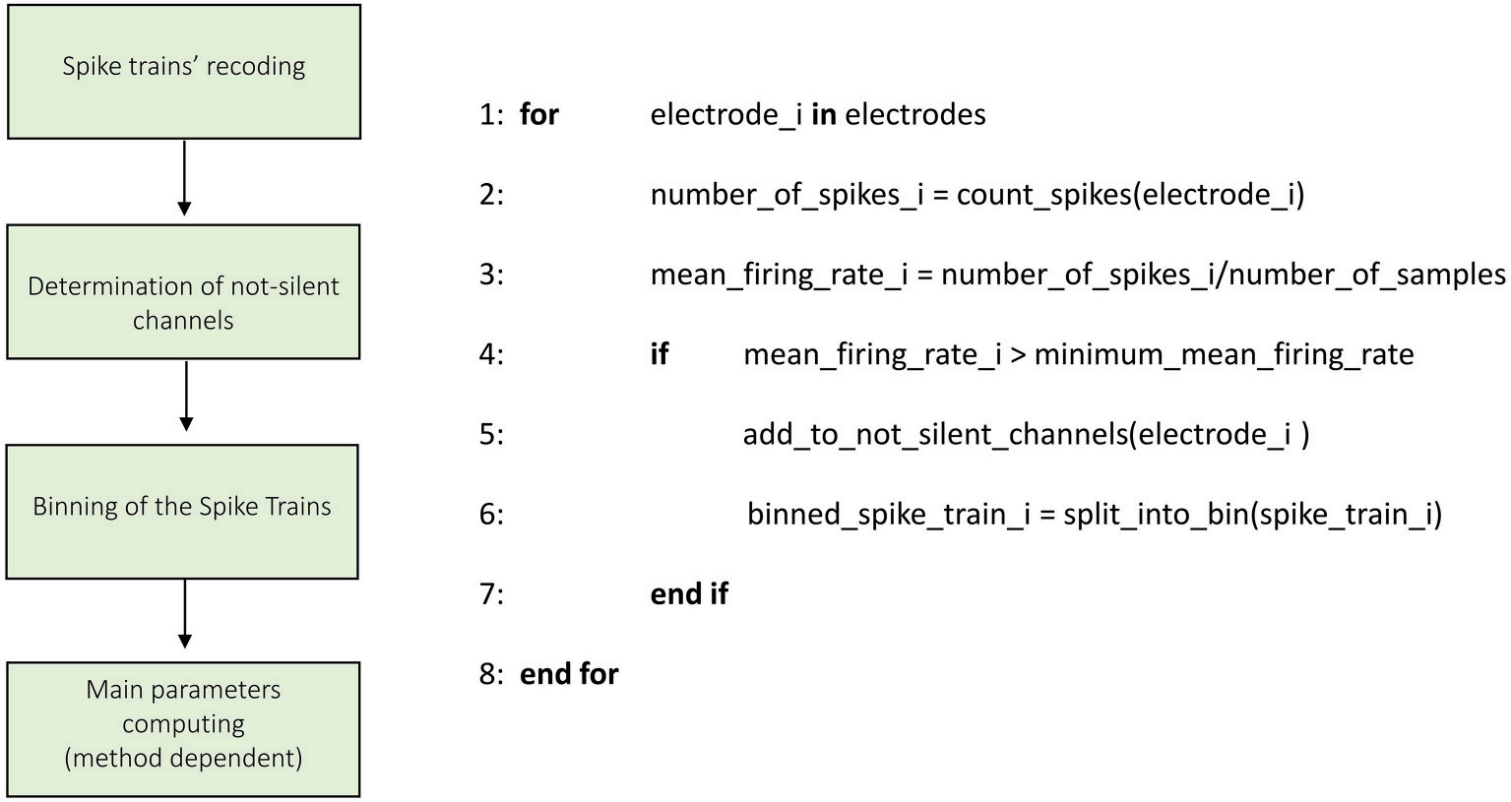
**Schematic representation and description of toolconnect's Initialization phase**. The Initialization phase, is the same for all the implemented algorithms.

**Figure 4:**
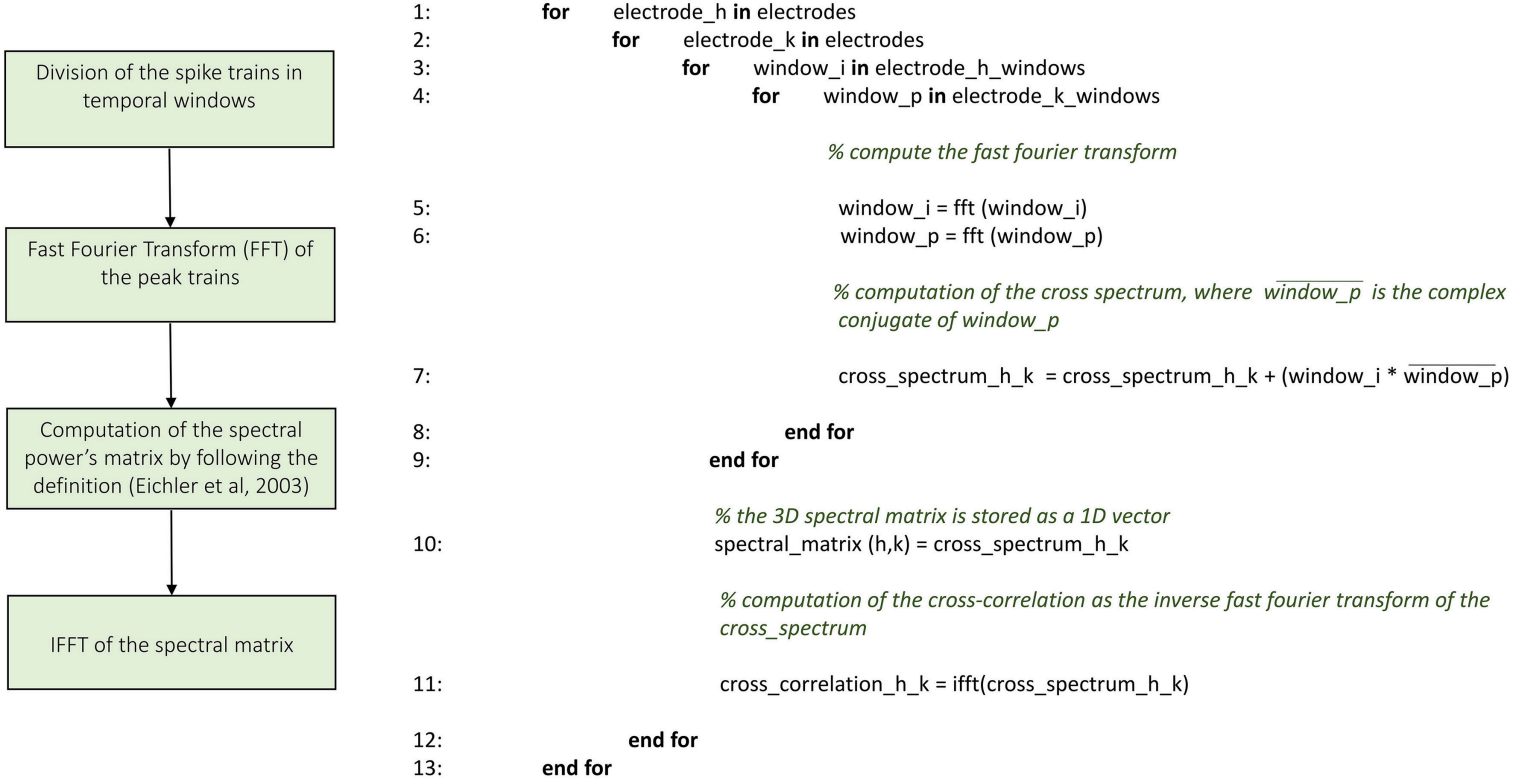
**Schematic representation and description of toolconnect's cross-correlation procedure in the frequency domain**.

**Figure 5:**
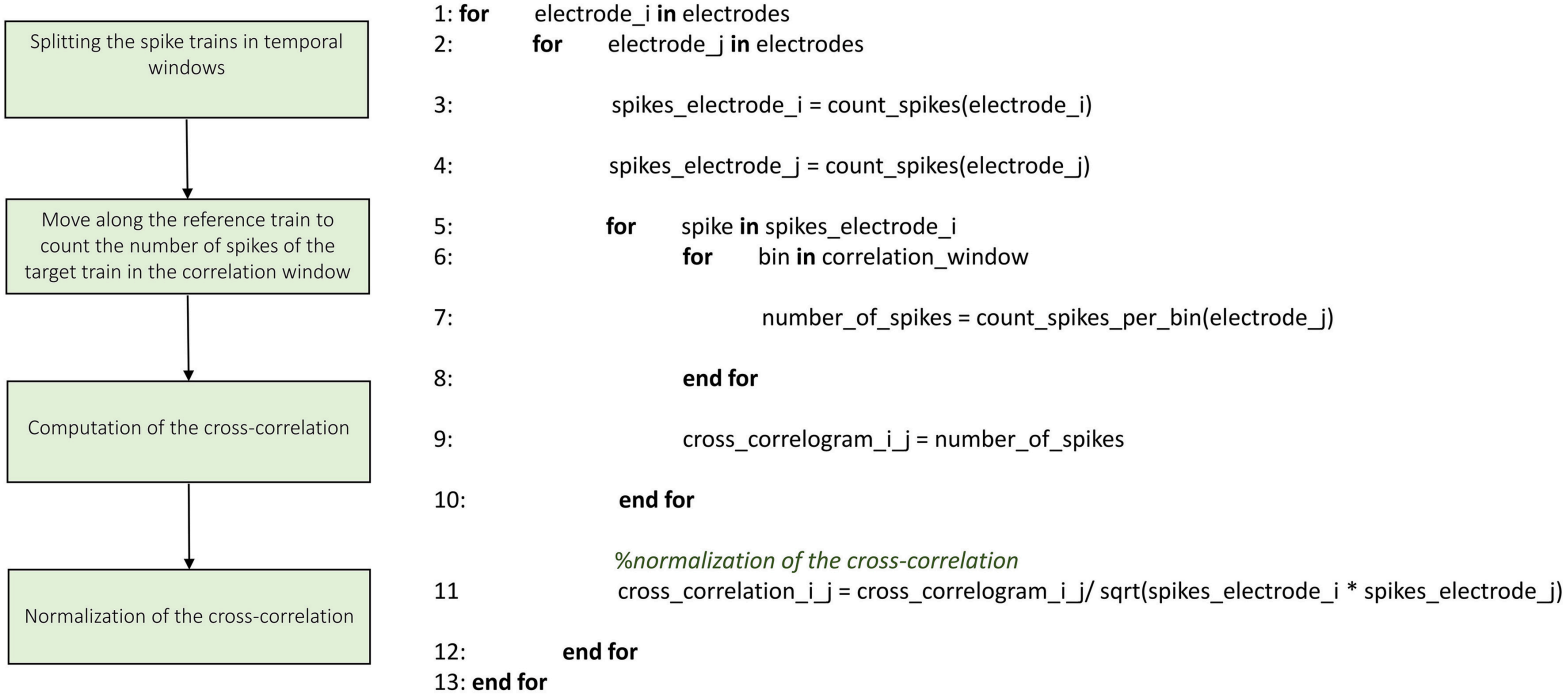
**Schematic representation and description of toolconnect's cross-correlation procedure in the time domain**.

**Figure 6:**
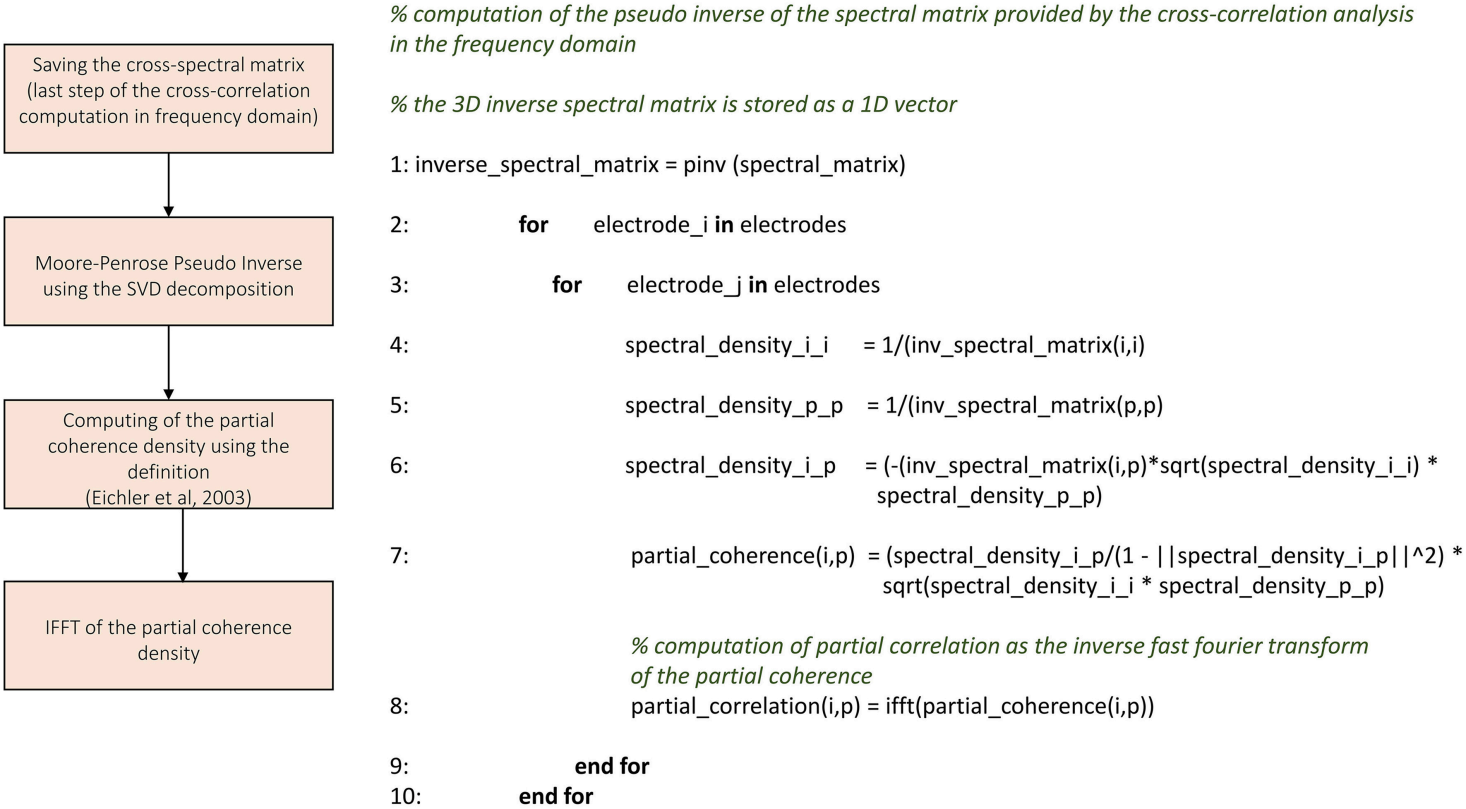
**Schematic representation and description of toolconnect's partial correlation procedure**.

**Figure 7:**
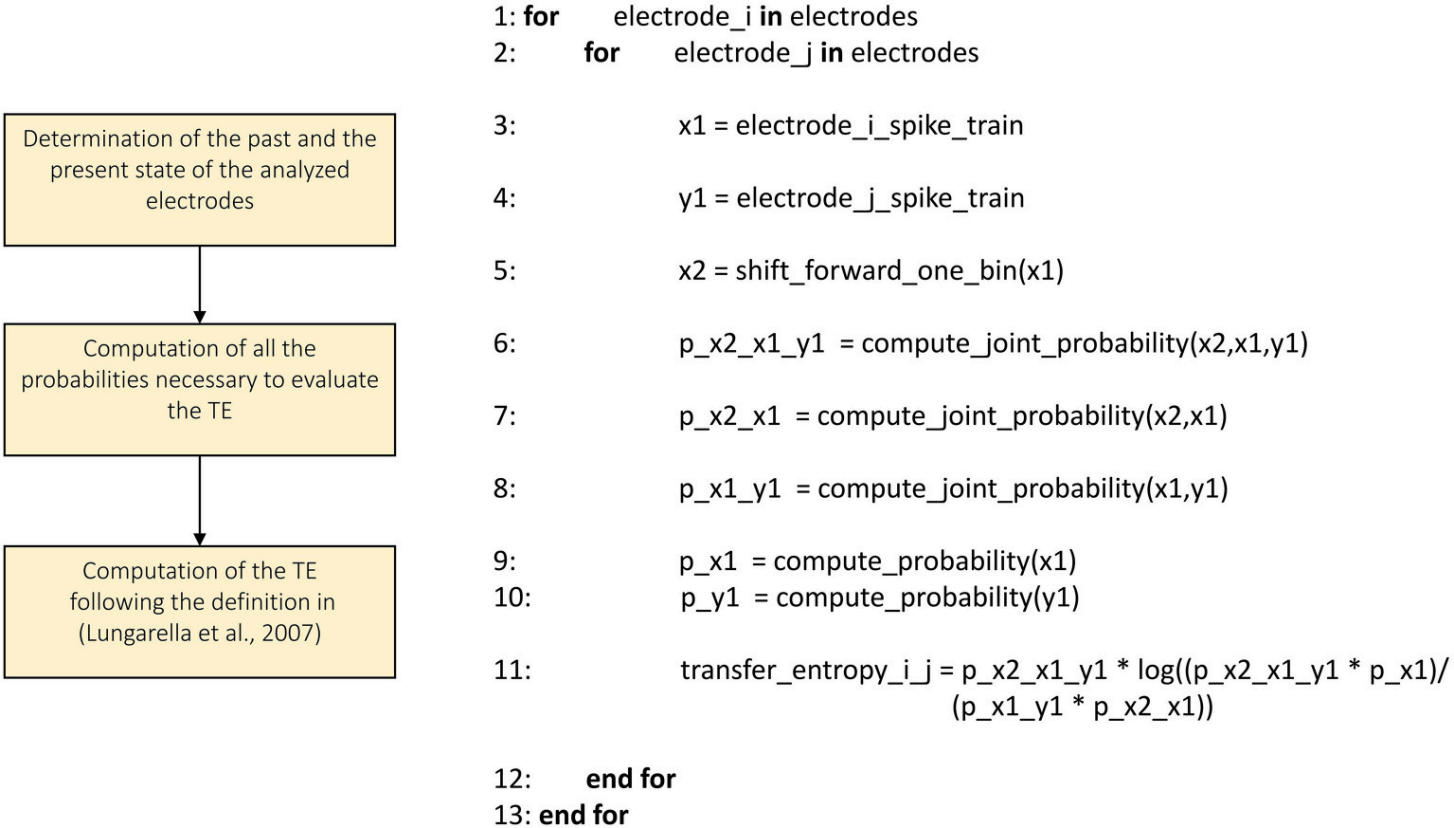
**Schematic representation and description of toolconnect's transfer entropy procedure**.

**Figure 8:**
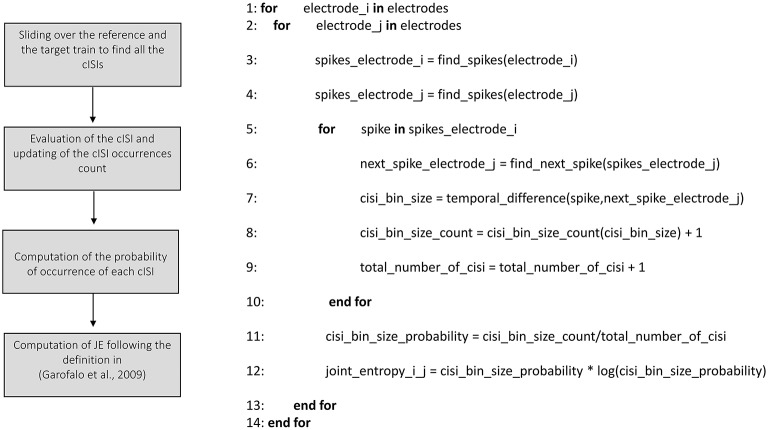
**Schematic representation and description of toolconnect's joint entropy procedure**.

Figures 3–**8** schematically represent and describe in the form of block diagrams and pseudocode the operations executed in the connectivity methods.

Figure 3 shows the schematic representation of the initialization process. The first operation is the recoding of the spike trains. As described in the previous section, ToolConnect's input data is represented by spike trains formatted as a sequence of the time stamps corresponding to the samples of occurrence of the spikes. In this part of the program, we format the spike trains as a sequence of number of samples elements, with a one if there is a spike in that sample, and a zero otherwise. This operation is necessary because the connectivity methods work on the complete samples' sequence of the spike trains. However, the spike trains formatted as described before require a huge amount of RAM to be stored. Moreover, ToolConnect is designed to be virtually independent (as a function of the hardware on which relays) from the size of the set of electrodes. Considering that recording systems with thousands of electrodes (Matsuda et al., 2003; Berdondini et al., 2009) are available, we were forced to find a solution for the large amount of RAM requested. We made use of the open source library of *Math.Net* (www.mathdotnet.com), that provides an implementation of the sparse matrix memory data storage strategy (i.e., a matrix where most of the elements are zeros, and only the non-zeros elements are memorized with the corresponding row and column indexes). Such a choice allows to minimize the amount of computational resources required by the correlation's algorithms and was motivated by the intrinsic sparsity of the spike trains acquired from *in vitro* neural network coupled to the MEAs. In fact, experimental recorded spike trains generally display a firing rate, which spans between 0.2 and 20 spikes/s, we can infer that in 10 min of recordings (sampling frequency 10 kHz) only in the 0.2% (approximately) of the samples a spike occurs. The second block permits the selection of the active electrodes. During an experimental acquisition, some electrodes could show no activity or very low firing rate values, meaning that they carry out a negligible amount of information. Thus, we offer the user the possibility to exclude these electrodes from the connectivity analysis, to decrease and optimize the RAM usage and the computational (running) time. According to this, we provide the user with the possibility to specify a minimum mean firing rate (MFR), below which an electrode is considered “silent” and discarded from further analysis; the default value is set to 0.1 spikes/s.

The third block consists in the binning of the spike train operations. Spike trains are split into bins of a defined size (specified through the graphical interface), and the binned sequences are used in the further analysis. Finally, the initialization procedure performs the computation of the main parameters necessary in the further analysis (different for the various connectivity methods) and the predisposition of the output's folders structure.

##### Cross-correlation

ToolConnect offers two independent implementations of the cross-correlation function, one in the frequency and one in the time domain. We implemented the cross-correlation algorithm in the frequency domain following the approach devised by Eichler et al. (2003). Figure 4 shows the schematic representation and description of the implemented cross-correlation procedure in the frequency domain. A dedicated graphical user interface allows the user to set the parameters for this analysis: the correlation window's temporal width, the bin size, the percentage of overlap between windows and the maximum temporal range around zero to look for the peak in the cross-correlogram (a necessary operation to build the connectivity matrix). After the initialization procedure, we divide each spike train into temporal windows (i.e., correlation windows). We considered the possibility to overlap two adjacent correlation windows while temporally moving over the spike trains, where the percentage of permitted overlap is one of the parameters described before. All the operations described in the following sections are executed considering the spike train's portion falling within each correlation window and iterating over the entire spike trains. First, we compute the Fast Fourier Transform (FFT) of the considered spike trains and then the cross-spectrum for each pair of electrodes multiplying the spectrum of the reference train by the complex conjugated of the target train's one. Finally, we determine the cross-correlogram as the Inverse Fast Fourier Transformation (IFFT) of the cross-spectrum. We normalize the cross-correlation (cf., Section Materials and Methods) dividing by the square product of the peak of the autocorrelation function of the analyzed electrodes. In this way, symmetric cross-correlation functions with a maximum value equal to one are obtained.

**Figure 9:**
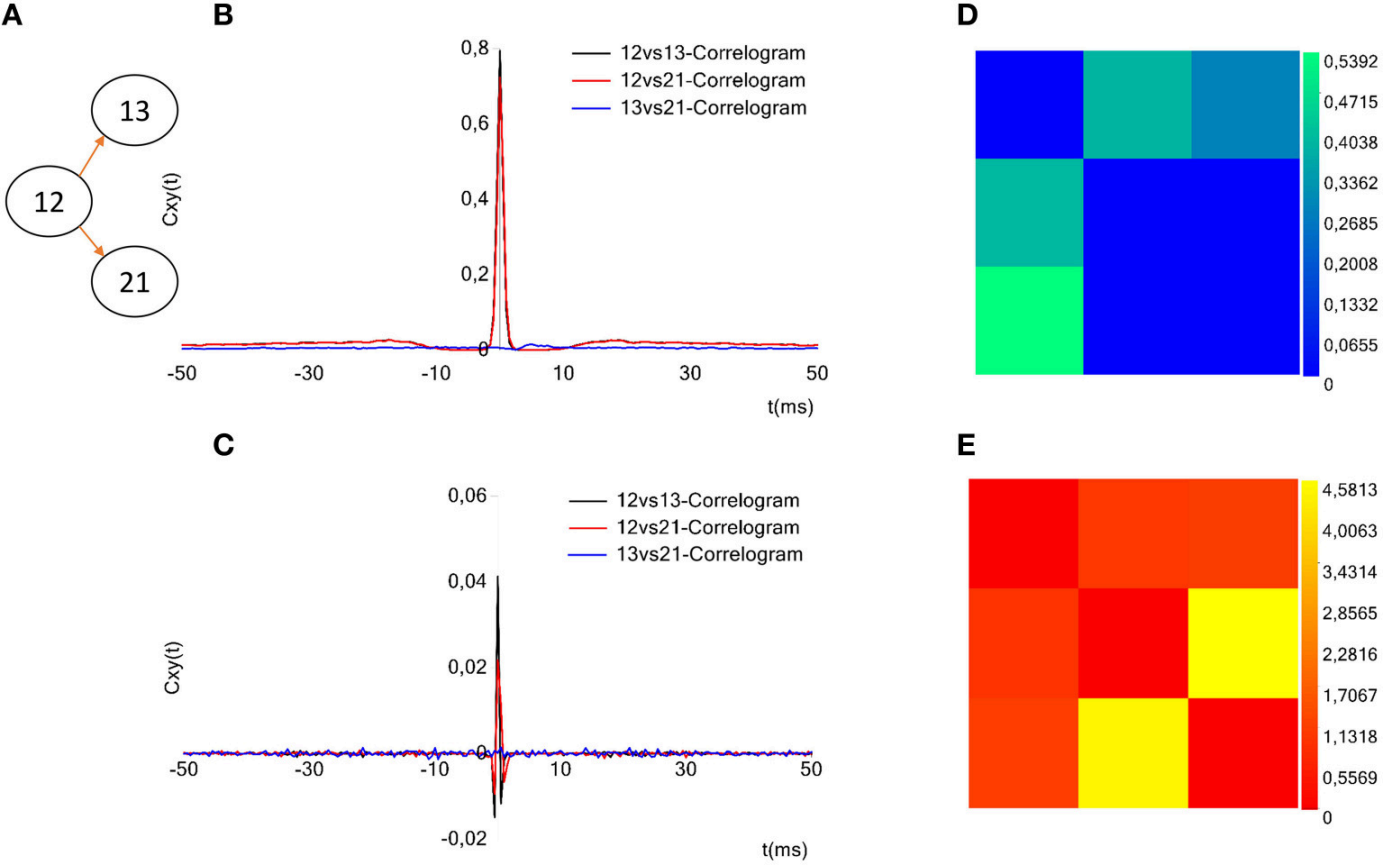
**Example of toolconnect's graphical output. (A)** Three neurons simulated neural network **(B)**, Cross-correlograms computed among the three electrodes. **(D)** Partial correlograms computed among the three electrodes. **(C,E)** Correspondent TE and the JE matrices. The peak appearing for the cross- and partial correlograms 12 vs.13 and 12 vs. 21 corresponds to a connection found between the aforementioned electrodes. In the same way, the TE presents the highest values for the couples (12, 13) and (12, 21) and the joint entropy shows the smallest values for the same couples; meaning that these methods found a connection between the aforementioned electrodes. Regarding the electrodes 13 and 21, instead, the absence of peak in the cross-and partial correlograms, the low values for TE and the high values for JE suggest the absence of a connection, as the sketch of the panel **(A)** shows.

The output of the cross-correlation analysis is one correlation file per electrode and the obtained connectivity matrices (cf., Section Cross-correlation and partial correlation connectivity matrices). The correlation file is a sequence of the cross-correlation functions computed by considering the correspondent electrode as the reference one, and varying the target electrode among the entire set of active electrodes; each cross-correlation function is preceded by the correspondent target electrode's index. The cross-correlation function gives the cross-correlogram values (**Figure 9B**) for each bin from –*T* to +*T* (where T is the correlation window's temporal width).

The implementation of the cross-correlation algorithm in the time domain (Figure 5) merges the classical approach of Knox (1981) and Rieke et al. (1997) and the one devised by Eytan et al. (2004). Figure 5 shows the schematic representation and description of the implemented cross-correlation procedure in the time domain. As for the frequency domain, a dedicated form provided through the GUI permits to set the parameters for this analysis: the correlation window's temporal width, the bin size and the maximum temporal range around zero within to look for the peak in the cross-correlogram (in order to build the connectivity matrix, when requested). After the initialization procedure (described in the previous section) we divide each spike train into temporal windows (i.e., correlation windows), and each temporal window into bins (where its size is one of the main parameters set in the initialization section, as described before). Let us consider a reference train *x* and a target train *y*; for each bin containing spikes in the train *x*, we center a temporal window (i.e., correlation window) on the bin correspondent to the aforementioned spike in train *y*. At this stage, we count the number of spikes within each bin and we repeat the procedure for every spike of the reference train *x*, updating the number of spikes per bin; in this way, we obtain the cross-correlogram. Finally, we normalize the cross-correlogram by using the squared product of the total number of spikes in the correspondent trains of the two analyzed electrodes; the cross-correlation functions obtained after the normalization are symmetric and have maximum value equal to one.

It is worth noticing that the cross-correlation computation in the time and frequency domains produces equal results (**Figure 9B**).

##### Partial correlation

We implemented the partial correlation's algorithm using the approach proposed in Eichler et al. (2003). Figure 6 shows the schematic representation and description of ToolConnect's partial correlation procedure. The algorithm performs all the operations necessary to compute the cross-correlation results in the frequency domain (cf., Section Cross-Correlation) before starting the computation of the partial correlation. Indeed, the partial correlation algorithm requires the cross-spectrum matrix as an input argument. The cross-spectrum matrix is a 3D matrix formatted as *n* × *n* × *nfft*, where *n* is the number of electrodes involved in the analysis and *nfft* is the number of points used to compute the FFT. We applied a vectorization procedure (i.e., we stored the 3D matrix as a 1D vector) to this matrix to significantly decrease the computational time and to minimize the RAM usage. Then, we computed the Moore-Penrose pseudo inverse of the cross-spectrum matrix, following the Singular Value Decomposition (SVD) provided by the open source library Math.Net Iridium (www.mathdotnet.com). In practice, we divided the cross-spectrum matrix with respect to the FFT data points, obtaining *nfft* matrices of size *n* × *n*; we inverted separately each of these matrices to obtain the final cross-spectrum pseudo inverse. At the next stage, we computed the partial coherence density following the definition (cf., Section Materials and Methods); then, we determined the partial correlation function among the analyzed electrodes by computing the IFFT of the partial coherence density. We considered a scaled version of the partial correlation (Eichler et al., 2003), obtained by dividing the partial correlogram relative to two electrodes with the squared product of the two autocorrelation peaks (as described for the cross-correlation). In this way, we obtain symmetric partial correlation functions (Poli et al., 2016). The output format of the partial correlation procedure is analogous to the cross-correlation's one; thus obtaining one correlation file per electrode and the connectivity matrices (cf., Section Cross-correlation and partial correlation connectivity matrices). The correlation file is a sequence of the partial correlation functions computed by considering the correspondent electrode as the reference one, and varying the target electrode among the entire set of electrodes; each partial correlation function is preceded by the correspondent target electrode's index. The partial correlation function saved in these files gives the partial correlograms values (**Figure 9C**) for each bin from –*T* to +*T* (where T is the correlation window's temporal width); thus, its length depends on the parameters chosen at the beginning of the analysis.

##### Cross-correlation and partial correlation connectivity matrices

The output of both partial and cross-correlation procedures are three *n* × *n* connectivity matrices (CM), where *n* is the number of the analyzed electrodes. The matrices are: (i) the symmetric CM, (ii) the directional CM and (iii) the synaptic delays matrix. The (*i, j*) element of the symmetric CM corresponds to the peak value (i.e., the maximum) of the cross-correlation function evaluated around the zero within the temporal range set by the user at initialization. This matrix is symmetric: both cross- and partial correlation functions are symmetric by definition (cf., Section Cross-correlation and Partial correlation). On the other hand, the directional CM is computed by searching for the peak only in the positive range of the correlation window; in this way, it is possible to determine the direction of the detected connections. Both cross- and partial correlation store information on the connection's direction in the delay of the peak with respect to the center of the correlation window. Let us consider the correlation window temporally centered on the zero; if the peak falls in the negative portion of the correlation window (i.e., if the peak is found on the left with respect to the center), the connection is directed from *j* to *i* (meaning that the electrode *j* is pre-synaptic for the electrode *i*). An opposite situation corresponds to the peak found in the correlation window's positive portion (i.e., if the peak is found on the right with respect to the center). If the peak falls in the central bin, no indications can be obtained about the direction of the detected connection. The (*i, j*) element of the delay matrix represents the peak's delay (expressed in milliseconds) between the electrode *i* and the electrode *j*, with respect to the zero.

##### Transfer Entropy

We implemented the Transfer Entropy (TE) algorithm following the definition provided in Gourévitch and Eggermont (2007) and Lungarella et al. (2007). Figure 7 shows the schematic representation and description of the transfer entropy procedure. The parameters to be set are the bin size in order to split the analyzed spike trains into temporal bins and the minimum mean firing rate to exclude the silent electrodes. Then, for each electrode pair, we evaluate the probability values necessary to compute the first order TE according to its definition (cf., Section Transfer entropy). Let us consider a reference train *x* and a target train *y*, the probability values to compute are: *P*(*x*_2_*|x*_1_)*, P*(*x*_2_*|x*_1_,*y*_1_), and *P*(*x*_1_,*y*_1_), where *x*_2_ and *x*_1_ are the past and the present state of the train *x* respectively, while *y*_1_ is the present state of the train *y*. By using the definition of conditional probability we compute the probability *P(x*_2_*|x*_1_,*y*_1_*)* as *P(x*_2_,*x*_1_,*y*_1_*)/P(x*_1_,*y*_1_*)* and analogously the probability *P(x*_2_*|x*_1_*)*. Practically, considering all the bins of the spike trains, we computed the aforementioned joint probabilities by counting the number of occurrences of every possible outcome of the two events: simultaneous spike in both of the trains, a spike in only one of the trains, and no spike in both of the trains. Then, we computed the requested probabilities by dividing the number of occurrences of each event by the total number of bins, obtaining the frequency of occurrences. Finally, we obtained the TE following the definition given in Lungarella et al. (2007).

##### Joint Entropy

We implemented the Joint Entropy (JE) algorithm following the definition reported in Garofalo et al. (2009), based on the determination of the cross-Inter-Spike-Intervals (cISI) histogram (cf., Section Joint entropy). Figure 8 shows the schematic representation and description of the implemented joint entropy procedure. Let us consider a reference train *x* and a target train *y* split in bins (as explained in the previous sections); the cISI is the temporal difference between a spike in the train *x* and a subsequent spike in the train *y*, expressed in bins. To build a cISI histogram, we need to evaluate the probability of occurrence of each possible cISI. Thus, we need to evaluate the frequency of occurrence of each cISI (that is the total number of times we find a cISI of size equal to a specific number of bins divided by the total number of cISI). More specifically, we have to consider each bin containing spikes in the reference train. Once we find such a bin, we start to move on the target train until we find another bin containing spikes. At this point, we evaluate the temporal difference between the two bins (i.e., the cISI), we update the cISI of that size count (to compute the number of occurrences) and the total number of cISI we found (for estimating the probability). To decrease the computational time, we restricted the number of bins to search for spikes in the target train. Finally, we use the probability of occurrence of each cISI of size *k*-bins (*p*(*cISI*_*k*_)) to determine the Joint Entropy according to its definition.

##### Multi-threading implementation

ToolConnect is developed as a Multiple Document Interface (MDI) windows form application. The parent form is a frame in which the implemented computational and graphical tools' interfaces are opened and displayed. Each of these interfaces is implemented as a windows form independent from the others. A friendly GUI and a complete set of feedback information are the fundamental principles in the toolbox's implementation. Accordingly, each windows form relies on a multi-thread implementation: some running threads are delegated to the update of the graphical interface while some others execute the effective code of the selected connectivity method. When performing functional connectivity analysis, the need of a large amount of available RAM and the high computational time are the two main issues to deal with efficiently. The multi-threading environment permits the various connectivity methods to be performed simultaneously on different threads of the different available CPUs, significantly reducing the requested computational time. Finally, by changing the application configuration settings file (“App.config”) we removed the default block on the allocation of very large object. In this way, we allowed a dynamic memory allocation strategy that depends on the available RAM.

#### Graphical section

In this Section, we describe the characteristics and the implementation of the graphical tools embedded in ToolConnect. We focused on the user-friendliness by including the possibility to view and graphically analyze previously obtained results. The main graphical tools embedded in ToolConnect are the cross- and partial correlograms plot and the computation, thresholding and plotting of the connectivity matrices and the connectivity graphs.

##### Plot correlograms

ToolConnect offers a dedicated interface to plot the cross- and partial correlograms. As for the selection of the electrodes to analyze (cf., Section Initialization section), if the acquisition system is successfully identified, it is displayed on the GUI and the user can graphically select the electrodes to plot the correlograms for (Figure 2, bottom left, blue box). Currently, this feature is available for some of the main commercial acquisition systems: the 60, 120, and 252 electrodes of MCS, the 64 electrodes of Panasonic MED64 system and, the 4096 electrodes of the 3Brain system. However, the developer can extend these features to other commercial acquisition systems in a very simple way (cf., Section Initialization section). Alternatively, it is possible to digit in the provided textboxes the numeric code of the electrodes to be considered in the correlogram's plot. A simple menu guides the user through all the tools designed for the customization of the correlograms' plot: line color, line width, axis range and the multiple correlograms plotting on the same graph. ToolConnect also allows the user to save the correlograms at a desired resolution. Figure 9B shows an example of a cross-correlogram for the simple neural circuit represented in Figure 9A. Figure 9C shows the correspondent partial correlogram; the peak visible in the correlograms (blue and red lines) represents a connection detected between the corresponding electrodes. Finally, Figures 9D,E show the correspondent TE and JE matrices.

##### Computation and plotting of the connectivity matrix

ToolConnect provides a dedicated interface to manage the Connectivity Matrix (CM) computation and visualization. It is possible to compute and plot the CM directly from the cross- or partial-correlograms. The main parameters to be set for computing the CM are the bin size, the sampling frequency, the size of the correlation window and the number of electrodes involved in the cross- or partial-correlation performed analysis. Another possibility is to plot an already existing CM. The CM is plotted in false colors; this means that the element (*i, j*) of the plotted matrix has a color code proportional to the value of the element (*i, j*) of the CM, proportional to the strength of the connection detected between the electrodes *i* and *j*.

##### Thresholding of the connectivity matrix

A crucial step to infer the functional connectivity of a network is the thresholding procedure. Once that a connectivity method provides a CM, the highest values are expected to correspond to the most likely connections. A procedure to select the strongest links is necessary since the connectivity methods provide a value for each electrode pair, independently from the existence of a direct or an indirect link, a simply random co-activation or a noisy link. Thus, we provide the user with the possibility of thresholding the CM to eliminate those values that are relative to noise and not to statistically significant connections. We can distinguish several thresholding procedures, with different level of complexity. There are complex thresholding methods based on the shuffling procedures (Grun and Rotter, 2010) which destroy the possible correlation between the pairs of electrodes by managing the spike trains in different ways, obtaining independent data (i.e., surrogate data). The simplest and computationally least expensive (i.e., fastest) thresholding procedure, instead, is the use of a hard threshold, defined in function of the CM's values. This thresholding procedure is strongly dependent on the distribution of the CM's values. Shuffling techniques are more precise and less heuristic, but they are computationally expensive. Thus, when dealing with the problem of thresholding the CM, it is important to choose the best compromise between reliability and computational time, depending on what one wants to claim from that specific analysis (Poli et al., 2015). In the current version, ToolConnect implements both a hard threshold and a shuffling approach.

*Hard threshold*. We implemented a simple heuristic procedure choosing a threshold equal to μ + *n* · σ, where μ and σ are the mean value and the standard deviation of the non-zeros CM's elements, respectively. ToolConnect provides the user with the possibility to specify the parameter *n* of the equation in order to compute and plot the Thresholded CM (TCM). Together with the TCM, we compute and plot the correspondent connectivity graph, which is the set of nodes and branches individuated in the matrix. Nodes represent the electrodes involved in the analysis, while links represent the functional connections found among them. It is also possible to modify the value of the thresholding parameter and to re-compute and re-draw both the TCM and the connectivity graph in real time. At present, the connectivity graph rendering is implemented only for the acquisition systems cited in the previous section.

*Shuffling thresholding procedure*. We implemented the *spike time dithering* surrogate data generation. Spike time dithering randomly displaces each individual spike within a short time window around its original position. This procedure destroys the exact timing of the spikes and, in consequence, the temporal relations between spikes of simultaneously observed neurons. We implemented the spike time dithering using the class *random* from the standard library.

**Table:** 

1:	**for** spike_i **in** electrode_spikes
2:	**for** surrogate_j **in** number_of_surrogates
	% generation of a random number within a
	temporal window of width w
3:	displaced_spike_i = random.next (spike_i - w,
	spike_i + w)
4:	**end for**
5:	**end for**

**Figure 10:**
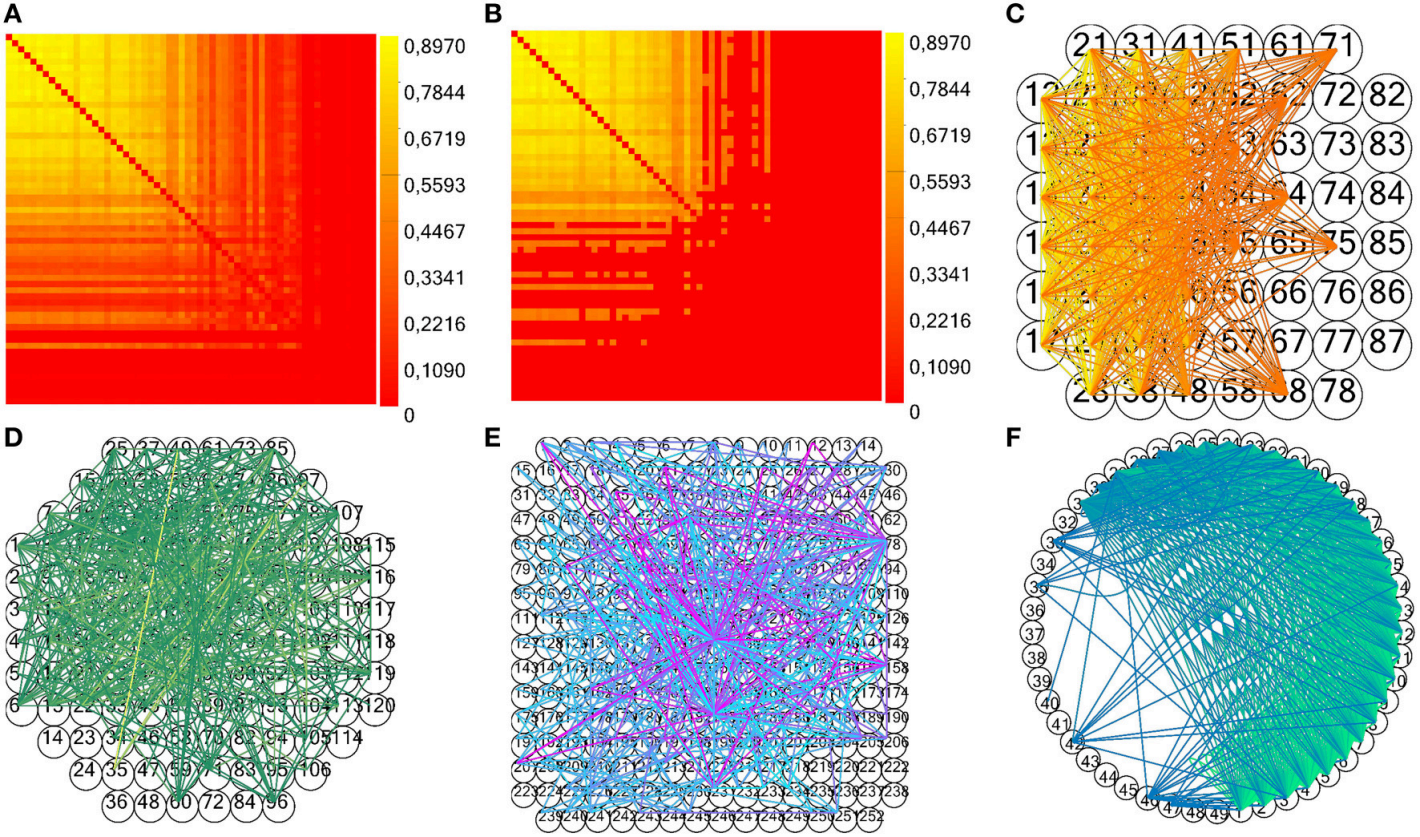
**Example of toolconnect's CM and connectivity graph. (A,B)** Examples of CM and TCM (obtained with *n* = 1) for a simulated network with medium average degree of connections (60 neurons, 30 mean connections per neuron). **(C–E)** Connectivity graph for the acquisition system MEA 60, 120, and 252 from Multi Channel Systems (MCS) respectively. **(F)** Example of unknown format (49 electrodes extracted from the aforementioned simulated neural network).

In particular, the method *next* of the class *random* generates a random number within an interval passed as argument. In the above pseudocode, *electrode_spikes* is the vector of the electrode's time stamps, while *w* is the temporal window's width in which we displace the spikes. We execute this operation for each of the surrogate to be generated (*number_of_surrogates*), in parallel, displacing all the spikes within the recorded spike trains. The parameters *w* and *number_of_surrogates* are set by the user through the GUI. We also implemented a significance statistical test to validate the estimated connectivity matrix: we compute the mean shuffled cross- (or partial) correlograms plus two times the standard deviation from surrogate data and compare the peak value to the estimated one in order to validate a connection (Grun and Rotter, 2010). For TE and JE, we compute the mean TE (or JE) matrix plus two times the standard deviation from shuffled data, and compare each element to the correspondent estimated one. Figures 10A–C display an example of CM, TCM and the correspondent connectivity graph relative to a 60 electrodes device. In Figures 10D,E, we can see the connectivity graph relative to 120 and 256 MCS acquisition systems. In case of unknown acquisition systems, the electrodes are arranged in a circular shape with straight and curve lines indicating the functional links among them (Figure 10F).

### Computational performances

We evaluated TOOLCONNECT's performances using two different metrics: the capability to correctly detect the connections among the analyzed electrodes (i.e., computational accuracy) and the running time (i.e., computational efficiency). We tested ToolConnect's performances on a PC provided with CPU Core i7 2.5 GHz and 16 GB of RAM hardware.

#### Efficiency and accuracy of the methods

To evaluate the performances of the developed methods in terms of “reconstruction of the original synaptic connectivity” (i.e., computational accuracy), we performed tests on a computational neuronal network model with *a priori* known synaptic connectivity matrix (cf., Supplementary Materials). In this way, it is possible to verify whether a connection detected by the connectivity methods corresponds to a real one (true positive) or it represents a false positive. In the same way, it is possible to determine if a not detected connection is actually absent in the analyzed network (true negative) or it represents a false negative. We can compare the synaptic connectivity matrix with the functional connectivity matrix produced by ToolConnect's methods. In this way, a quantitative assessment of the accuracy of each connectivity method is obtained. We used the ROC curves (cf. Section Receiver Operating Characteristic (ROC) Curve) and the correspondent AUC indicator to analyze the accuracy performances (cf., Section Materials and Methods) of the connectivity methods. As described in the previous sections, the first operation performed by each connectivity method implemented in ToolConnect is represented by the spike trains' splitting into temporal bins; the bin size is one of the main parameters in functional connectivity analysis; it largely influences both the computational efficiency and accuracy (in terms of AUC). We performed a quantitative analysis of such a dependence, by using a 60 neurons *in silico* network with dynamics described by the Izhikevich equations (Izhikevich, 2003) and random connectivity. Each neuron's connection probability is equal to 0.02. We simulated 10 min of spontaneous activity (sampling frequency 10 kHz). More details about the computational model can be found in the Supplementary Material.

**Figure 11:**
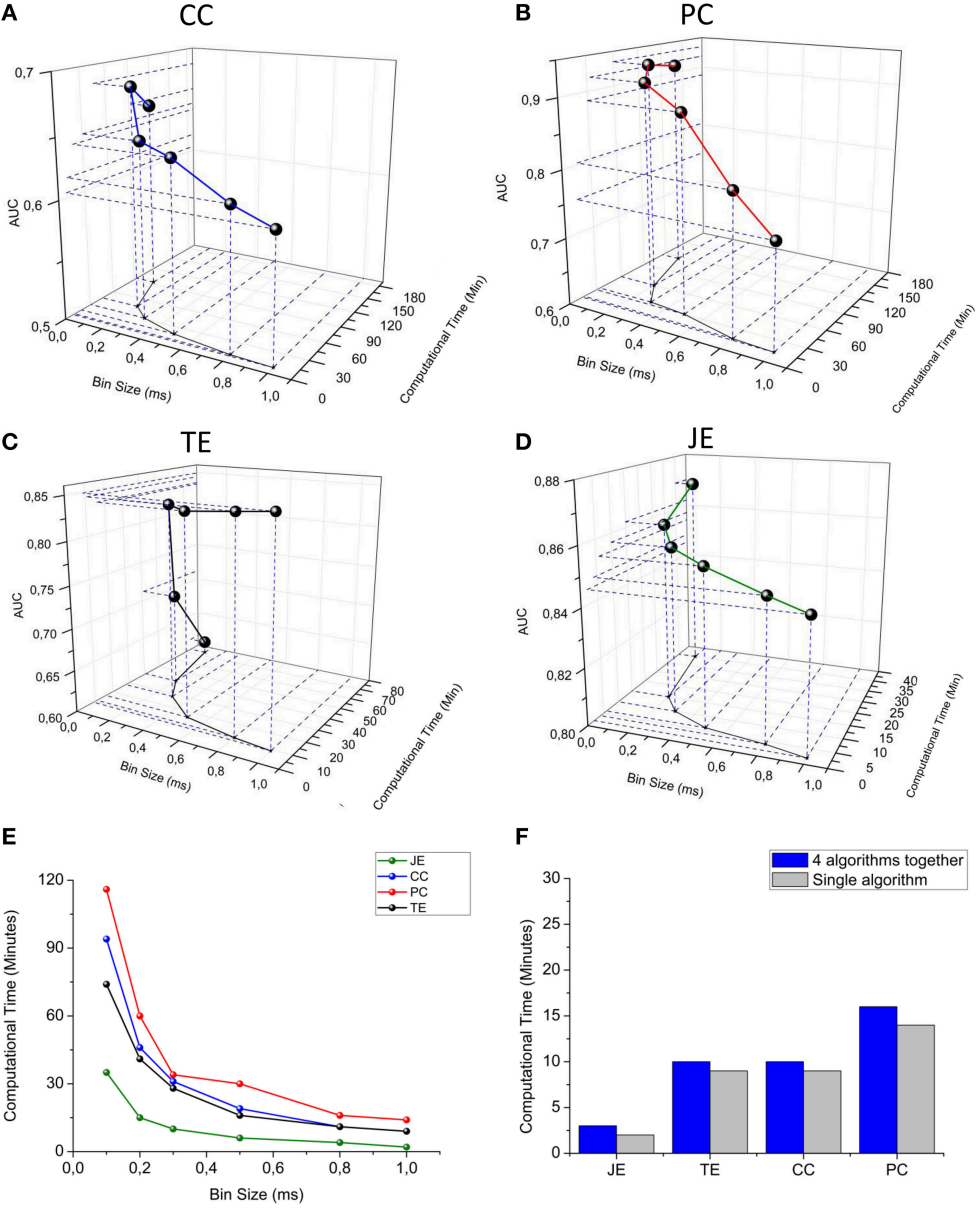
**toolconnect's performances**. In the first four panels, a three-dimensional representation of the dependence between bin size, computational time and AUC is presented. **(A)** Cross-correlation, **(B)** Partial correlation, **(C)** Transfer entropy, **(D)** Joint entropy. **(E)** Computational time vs. bin size graph for all the algorithms embedded in ToolConnect. **(F)** Comparison between the computational time required for each connectivity method when performed alone and when ran with all the other algorithms (multi-threading implementation).

We span the bin width of the connectivity methods from 0.1 to 1.0 ms. We observed that the computational time strongly decreases for all the connectivity methods when increasing the bin size; however, the AUC decreases when spanning from 0.1 to 1.0 ms, although such a value is always higher than 0.5 (i.e., the AUC correspondent to the random classifier). We can observe that Partial Correlation (Figure 11B) shows the best performances in terms of accuracy (AUC max = 0.94), but it has a higher computational time than information theory based methods (Figure 11E). Cross-correlation (Figure 11A) shows the worst overall performances (AUC = 0.69). Transfer Entropy's AUC increases significantly when changing the bin size from 0.1 to 0.3 ms, reaching a value equal to 0.84 and becoming approximately constant with respect to further increasing of the bin size (Figure 11C). This behavior arises from the fact that the sampling period in our simulations was equal to 0.1 ms, hence, such a bin size is not sufficient to correctly infer the dependence of the reference train activity from the target train's one. Joint Entropy (Figure 11D) exhibits the lowest computational time (for all the analyzed bin size's range) while still showing acceptable accuracy performances (AUC = 0.85). A good trade-off between the computational efficiency and accuracy was obtained by using a bin of 0.3 ms, correspondent to 34 min (total computational time) for the correlation methods and 20 min for the information theory based ones. Finally, we compared the computational time required to run each connectivity method alone with the one elapsed when all the connectivity methods run simultaneously. Figure 11F shows the results obtained by using a bin size of 1 ms (i.e., the bin correspondent to the minimum computational time); thanks to the multi-threading environment implementation, the computational times were almost comparable. The increasing running time spans from 18% in the case of TE up to 25% for the CC/PC and 33% for JE (i.e., the fastest algorithm).

### ToolConnect's application to experimental data

In the previous section, we tested and validated the ToolConnect's connectivity methods on an *in silico* neural network. In this section, we will present two examples of experimental applications to mature hippocampal assemblies coupled to standard 60 micro-electrodes MEA and to high-density 4096 APS chip. Details regarding the cell culture procedure and the experimental set-up can be found in the Supplementary Materials.

#### Spontaneous vs. stimulated networks

**Figure 12:**
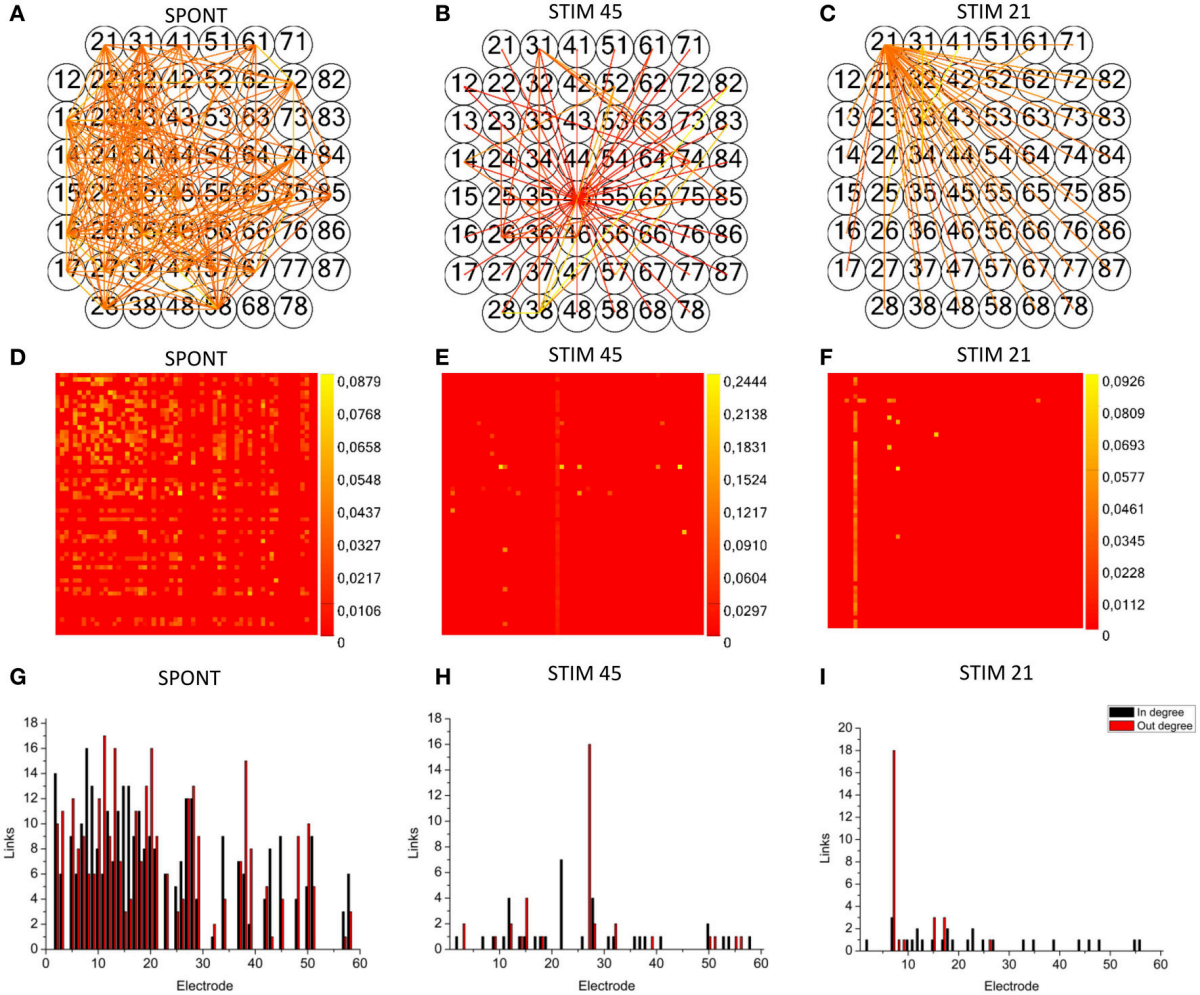
**toolconnect's application to mature hippocampal assemblies**. TOOLCONNECT's application to mature hippocampal assemblies. Cross-correlation algorithm was applied to hippocampal networks in spontaneous and stimulus-evoked conditions. **(A,D,G)** connectivity graph, connectivity matrix and degree distribution for spontaneous activity. **(B,E,H)** connectivity graph, connectivity matrix and degree distribution for stimulated activity (site of stimulation, electrode 45). **(C,F,I)** Connectivity graph, connectivity matrix and degree distribution for stimulated activity (site of stimulation, electrode 21).

**Figure 13:**
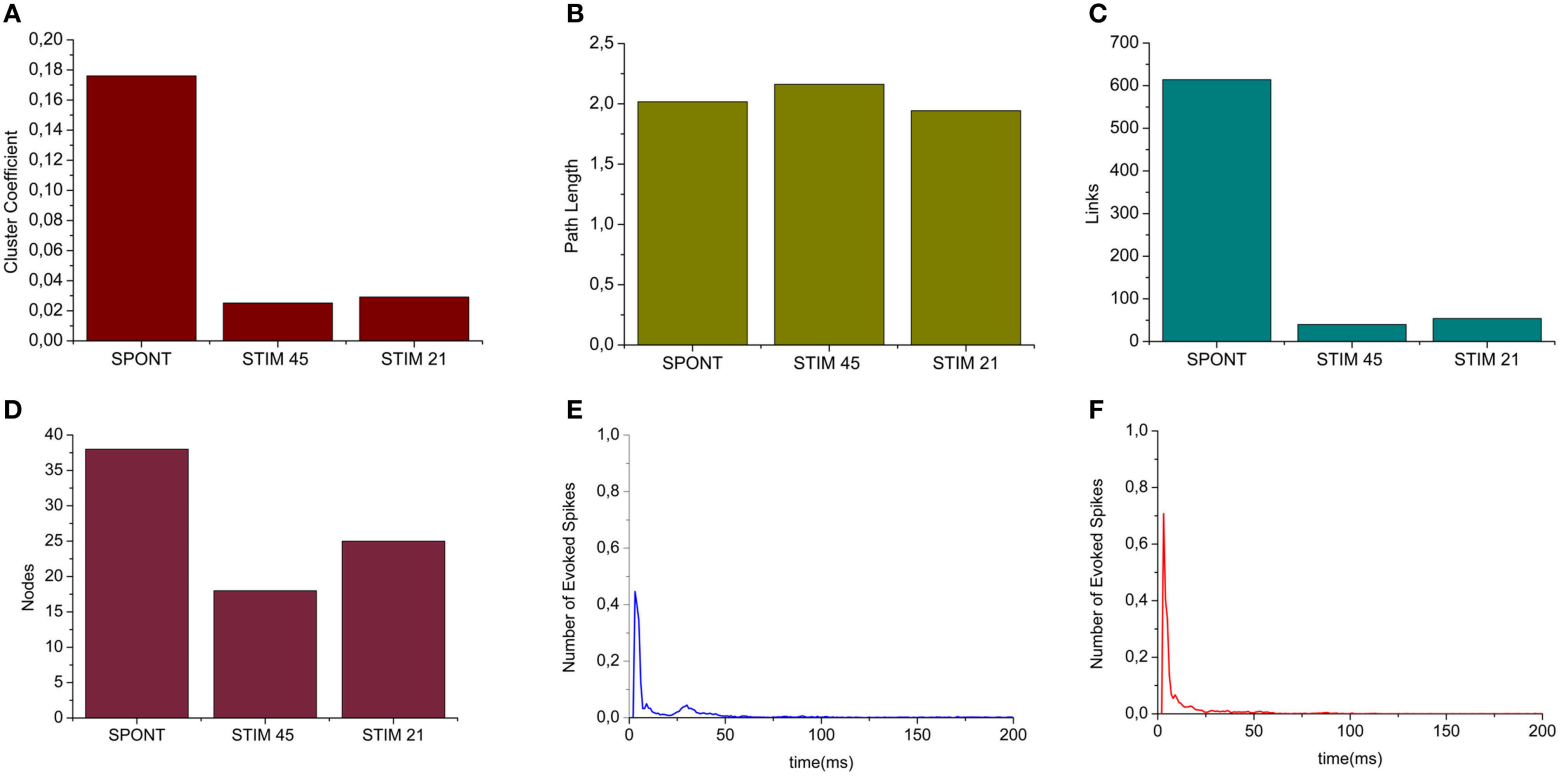
**Graph Theory's analysis of hippocampal neural assemblies. (A)** Cluster coefficient. **(B)** Path length. **(C,D)** Number of links and number of neurons found after the thresholding procedure, respectively. **(E)** PSTH relative to the stimulation of electrode 45. **(F)** PSTH relative to the stimulation of electrode 21.

We applied the cross-correlation algorithm (in the frequency domain) to cultured hippocampal neural networks during both spontaneous and stimulus-evoked activity. Electrical stimulation was performed by delivering bi-phasic voltage stimuli at the frequency of 0.2 Hz, with a peak-to-peak amplitude of 2.0V, from a single electrode. Analysis was performed on recordings lasting 10 min (sampling frequency of 10 kHz). Figure 12 shows the connectivity graphs and the connectivity matrices obtained for the spontaneous conditions (Figures 12A,D) and stimulation phases relative to a stimulation phase from channel 45 (Figures 12B,E) and 21 (Figures 12C,F). The 60 micro-electrodes MEA acquisition system we used, provides a blanking period of 250 μs after the stimulus, to avoid stimulation artifact that could be mistaken with spikes. In addition, during the spike detection, in order to be more conservative, we deleted further 2 ms of activity. Thus, the analysis of the functional connectivity we performed during the phases of electrical stimulation is relative to the electrophysiological activity starting from 2.25 ms after the stimulus delivery. All the connectivity graphs and matrices were obtained by using the hard-threshold algorithm setting a threshold equal to μ + *2*·σ, where μ and σ are the mean value and the standard deviation of the CM's elements, respectively (cf. Section Hard threshold). After the thresholding procedure, during spontaneous activity, we detected more links (about 90%) than during the stimulated activity (Figure 13C). During spontaneous activity, the detected functional links involve a larger number of electrodes (about 53%) than to the stimulated one (Figure 13D). However, the correlation values obtained for the spontaneous activity are lower but more homogeneous than the values correspondent to the stimulated one (maximum values' difference of 0.16, standard deviation 0.008 vs. 0.045 for spontaneous and stimulated activity respectively, Figures 12D–F). We also evaluated the in- and the out-degree distribution. We observed that during spontaneous activity, the in- and the out-degree for the different electrode were almost equally distributed among the active electrodes (Figure 12G). During stimulus-evoked activity, the stimulated electrode showed an increased number of outgoing connections, reaching a difference of 14 links with the other electrodes (Figures 12H,I). In the case relative to the stimulation of electrode 21, we had only one electrode with more than 3 outgoing connections. When stimulating the electrode 45, we found only one electrode with more than 4 outgoing-ingoing connections. In the spontaneous condition, instead, there were 35 and 28 electrodes with an out-degree of connections greater than 3 and 4 links, respectively. Finally, we used two metrics from graph theory (namely, cluster coefficient and path length) to evaluate the topological characteristics of the neural networks, in the two different experimental conditions. Figures 13A,B show the results relative to the cluster coefficient and path length, respectively. We observed that the path length is not affected by the electrical stimulation (i.e., same values for the spontaneous and the stimulus-evoked activity). On the other hand, we found a higher cluster coefficient for the spontaneous conditions than for the stimulated ones. This is probably due to the effect of the stimulation on the global network's dynamics: the stimulation empowers the outgoing connections from the stimulated electrode, thus, these connections become stronger, and correspond to higher cross-correlation values. We can notice that, the connectivity graph for the stimulation of the electrode 21 and 45 are quite different. In particular, the outgoing connections of the electrode 45, are weaker than the ones relative to the electrode 21 (Figures 12B,C). Moreover, the degree distribution relative to the electrode 45 shows a higher variability in the number of in-coming and out-going connections than the one relative to the electrode 21. Figures 13E,F show the population Post Stimulus Time Histogram (PSTH) relative to the two different sites of stimulation (i.e., the PSTH averaged over all the electrodes); we can observe that the number of evoked spikes by the stimulation of channel 45 (Figure 13E) is lower than the number correspondent to the stimulation of the electrode 21 (Figure 13F). It is possible to ascribe that the electrode 45 is less involved in the dynamics of the analyzed network with respect to the electrode 21.

#### Connectivity maps of hippocampal networks coupled to high-density MEAs

**Figure 14:**
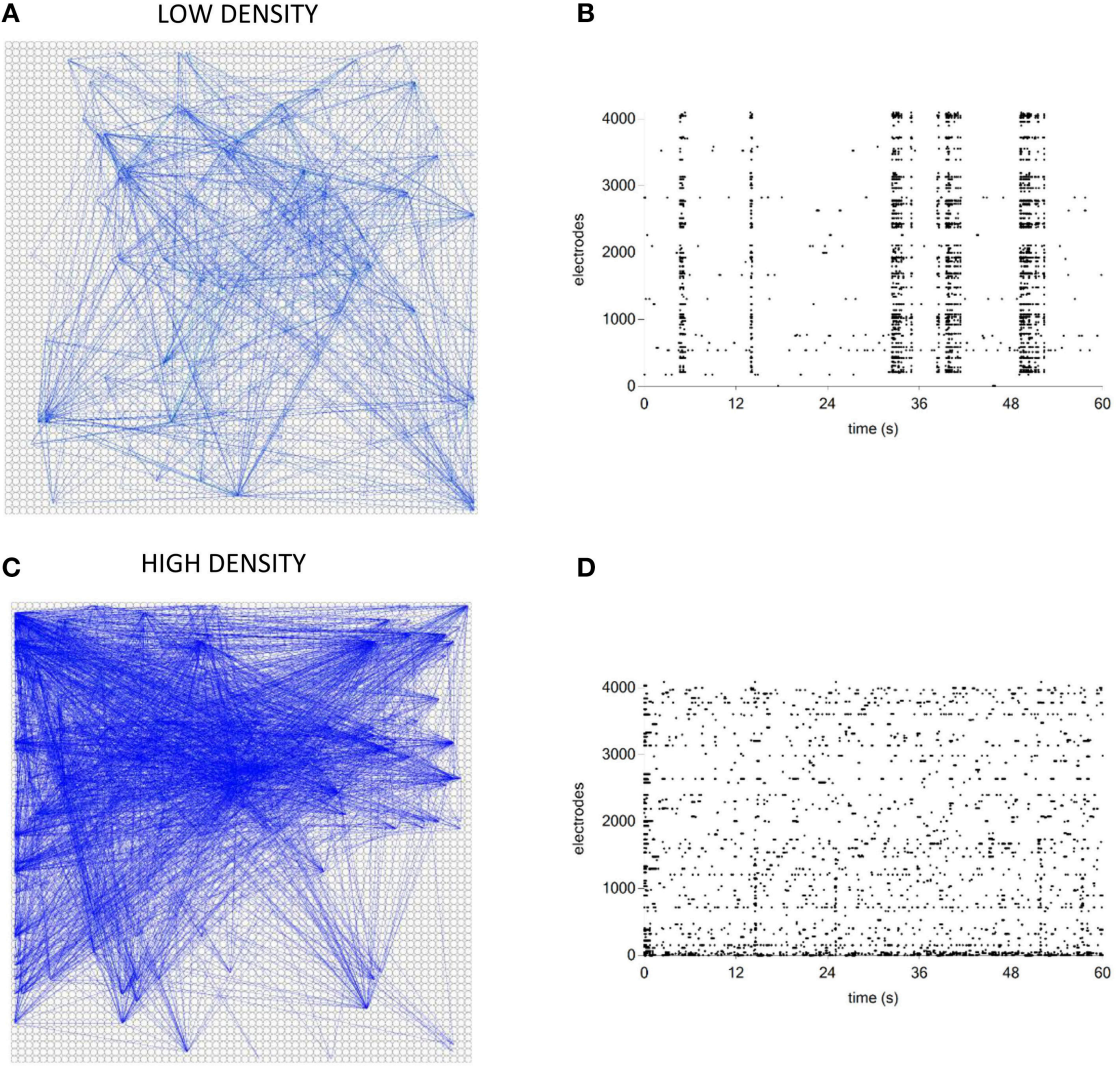
**Connectivity graphs relative to two hippocampal networks coupled to high-density MEAs. (A,C)** TE connectivity graph and raster plot for the low- density network. **(B,D)** TE connectivity graph and raster plot for the high-density network.

Finally, we tested the ToolConnect's capability to process data acquired by means of thousands of electrodes, trying to overcome the problems relative to the RAM usage management and the computational efficiency that we described in the previous sections. We analyzed two different hippocampal networks coupled to the APS recording system. The first one was a low-density culture (80–200 cell/mm^2^; Maccione et al., 2012), while the second one was a high-density culture (350–1200 cell/ mm^2^; Berdondini et al., 2009). Analysis have been performed on a recording chunk of 10 min (sampling frequency: 7022 Hz). Figure 14 shows the exhibited activity (Figures 14B,D) and the obtained functional connectivity maps by applying the TE algorithm on the low-density (Figure 14A) and on the high-density culture (Figure 14C). We chose to apply the TE algorithm (bin size of 1 ms), because of the large data size and the low computational cost of the method (cf., Section Computational Performances). At first glance, we can observe that the high-density network graph shows a higher number of links (15,726) and nodes (234) than the low-density correspondent one (5441 links and 130 nodes). The highest number of links in the high-density network is justified also by the dynamics, qualitatively displayed in the corresponding raster plots (Figures 14B,D): the low-density culture's firing rate (0.42 ± 0.36) is lower and more homogeneous than the high-density one (0.82 ± 2.41), which presents also a greater degree of random spiking activity.

## Discussion

Nowadays, the estimation of the functional connectivity in large-scale neuronal networks is a fundamental issue to understand the emergent network dynamics. In the panorama of the “neuronal connectivity literature,” several studies have been performed to estimate functional connections at different level of complexity, starting from small/large *in vitro* circuits, up to macro brain areas (Sporns, 2013). In this work, we presented a computationally efficient toolbox to estimate and characterize functional connectivity in *in vitro* neuronal networks coupled to MEAs. ToolConnect is an open source software, which implements correlation and information theory based algorithms to infer functional connectivity by analyzing the peak trains of spiking neuronal signals. It has been implemented as a standalone windows GUI application, using C# programming language with Microsoft Visual Studio based on .NET framework 4.5 development environment. In this work, we started with a detailed description of the software architecture. Then, we tested the software's computational performances in terms of efficiency (computational time) and reliable reconstruction of functional networks (computational accuracy). Currently, ToolConnect has been tested over two commercial acquisition systems: the 60/120 electrodes MCS MEA system, and the 4096 electrodes 3Brain APS system showing good overall performances. However, thanks to its flexibility, ToolConnect can be easily adapted to other acquisition systems. It is worth noting that in the current version of the software, the functional connectivity analysis can be performed only on spike trains data (i.e., point process). Indeed, allowing the analysis of time series data is an important task we are planning to deal with in the future.

Finally, it is important to underline that ToolConnect is available to the scientific community and it has been designed to be adapted, modified and extended by the interested researchers.

## Information sharing statement

ToolConnect is available on INCF-Software Center (http://software.incf.org/software/toolconnect).

## Author contributions

VP developed the functional connectivity methods, implemented the toolbox, and wrote the manuscript. DP and SM contributed to develop the connectivity methods. AG contributed in the manuscript writing and editorial revisions. PM contributed assisting and coordinating all the development, data analysis, manuscript writing procedures, and editorial revisions.

### Conflict of interest statement

The authors declare that the research was conducted in the absence of any commercial or financial relationships that could be construed as a potential conflict of interest.
