# Corrigendum: ToolConnect: A Functional Connectivity Toolbox for *In vitro* Networks

**DOI:** 10.3389/fninf.2017.00037

**Published:** 2017-06-14

**Authors:** Vito P. Pastore, Daniele Poli, Aleksandar Godjoski, Sergio Martinoia, Paolo Massobrio

**Affiliations:** ^1^Neuroengineering and Bio-Nano Technology Lab, Department of Informatics, Bioengineering, Robotics, System Engineering, University of GenovaGenova, Italy; ^2^Institute of Biophysics, National Research CouncilGenova, Italy

**Keywords:** functional connectivity, *in vitro*, micro-electrode arrays, multi-threading, windows form application, neural networks, correlation algorithms, information theory algorithms

In the Section “Information Sharing Statement” of our original article, the webpage of the repository where we uploaded ToolConnect software has been discontinued. For this reason, the new Information Sharing Statement Section is:

ToolConnect is available on NITRC repository (https://www.nitrc.org/projects/toolconnect/).

The authors state that this does not change the scientific conclusions of the article in any way.

## Conflict of interest statement

The authors declare that the research was conducted in the absence of any commercial or financial relationships that could be construed as a potential conflict of interest.

