# Supplementary material for: ToolConnect: A Functional Connectivity Toolbox for In vitro Networks
Source: Front Neuroinform. 2016 Mar 30;10:13. doi: 10.3389/fninf.2016.00013 (PMC4811958; doi:10.3389/fninf.2016.00013)
Supplement: Supplementary file 1 [file DataSheet1.DOCX]

Supplementary Materials

**ToolConnect: a functional connectivity toolbox for *in vitro* networks**

Vito Paolo Pastore^1^, Daniele Poli^1^, Aleksandar Godjoski^1^, Sergio Martinoia^1,2^, Paolo Massobrio^1^

^1^ Neuroengineering and Bio-nano Technology Lab (NBT), Department of Informatics, Bioengineering, Robotics, System Engineering (DIBRIS), University of Genova, Genova - Italy.

^2^CNR - Institute of Biophysics, Via De Marini, 6, 16149 Genova, Italy

**1. *In vitro* cell cultures and experimental set-up**

Dissociated neurons were extracted from rat embryos and plated onto 60 planar TiN/SiN micro-electrodes (30 µm diameter, 200 µm spaced) or 4096 Active-Pixel Sensor (APS) at the density of 1500 - 2000 cells/mm^2^. In a specific sub-set of 4096 APS chips the density was lowered to 350-1200 cells/ mm^2^.The procedures were approved by the European Animal Care Legislation and by the guidelines of the University of Genova.

Micro-Electrode Arrays (MEAs) and APS were coated with adhesion promoting molecules (poly-D-lysine and laminin). Neurons were maintained in culture dishes, each containing 1 ml of nutrient medium (i.e. serum free Neurobasal medium supplemented with B27 and Glutamax-I) and placed in a humidified incubator having an atmosphere of 5% CO_2_ at 37 °C. Further details can be found in ([Pasquale et al., 2008](#_ENREF_6);[Berdondini et al., 2009](#_ENREF_1)).

Electrophysiological activity was recorded during the fourth week *in vitro* to allow the maturation of the network ([Chiappalone et al., 2006](#_ENREF_2)). The results reported in the main text come from two experimental set-up. The first one is based on the MEA60 System (Multi Channel Systems, MCS, Reutlingen, Germany) consisting of a mounting support with integrated 60 channels pre- and filter amplifier (gain 1200×) and a personal computer equipped with a PCI data acquisition board for real time signal monitoring and recording. The second one is based on the BioCam4096 system (3Brain, Landquart, Switzerland) and consists of 4096 square microelectrodes (side: 21 μm). Raw data were visualized and recorded with the BrainWave software application provided with the BioCam4096 platform.

**2. Computational model**

The neural network model implemented to test the performances of the connectivity methods of ToolConnect is made up of 60 synaptically connected neurons, whose dynamics is described by the Izhikevich equations ([Izhikevich, 2003](#_ENREF_3)). Excitatory and inhibitory neurons were modeled by means of the family of regular and fast spiking neurons ([Izhikevich, 2004](#_ENREF_4)), respectively. The ratio between excitatory and inhibitory neurons was set to 4:1 in according to the experimental findings ([Marom and Shahaf, 2002](#_ENREF_5)). These two neuron families were linked following a random connectivity scheme. The mean probability of connectivity of each neuron has been set to 0.02. Spontaneous activity was obtained by introducing a randomly distributed stimulation reproducing the effect of fluctuation in the membrane potential. Simulations were sampled at 10 kHz and last 10 min. Simulations were performed in Matlab environment (The Mathworks, Natick MA, USA).

**3. ToolConnect’s function library**

The connectivity methods as well as the graphical tools embedded in ToolConnect have required the development of an ad-hoc function library. In this section, we will briefly report the name and a summary for the most important implemented function.

*Compute_on_Npoints.* This function is used to obtain a vector with exactly *n* (where *n* is an input) elements from the vector passed as input. If the vector has less elements than n elements, the output vector is zero-filled; otherwise, it is truncated.

*Inverse.* This function returns the inverse of a given matrix. If the matrix is square, the function uses the LU factorization in order to compute the inverse of the matrix; otherwise, the function uses a QR decomposition.

*PInvPast.* This is our implementation of the Pseudo-Inverse of Moore-Penrose, it is based on the SVD decomposition; the singular values lower than a specific tolerance are discarded by the function, it is analogous to the MATLAB’s function *pinv*.

*Compute_Max_correlation_peak Connectivity_Matrix.* This function search for the peak in the cross- and partial correlograms. One of its inputs is a parameter that indicates the number of bins to looking for the peak with respect to the zero bin.

*Contains.* This function indicates if a vector contains a specific element passed to the function as input;

*Find.* This function returns the index of the element in the vector correspondent to a value passed as input (-1 if the value is not found into the vector).

*Nonzeros.* This function returns the vector passed as input without zeros.

*electrode_find_file_position.* This function is necessary to overcome the electrodes’ file sorting operated by windows. It returns the position of a given electrode (identified by its code) in the vector containing the name (as string) of all the electrodes.

*ind2sub.* This function returns the vector containing the equivalent row and column subscripts corresponding to the linear index passed as input, in the a matrix of size described by the input vector "size" (it is analogous to the MATLAB’s function *ind2sub*).

*Sum, Zero_Sum.* This function actually refers to 12 different sub-functions. It counts the number of one or zeros in vectors passed as input. It is possible to pass up to three binary vectors, counting all the possible combination of the assumed values (e.g., one in all the vectors, one only in a certain vector and so on). This justifies the necessity to write 12 different sub-functions.

*Repmat.* This function tile and replicate an array in a specific format. Both the array and the format are passed as input; it is analogous to the MATLAB’s function *Repmat*.

**4. References**

Berdondini, L., Imfeld, K., Maccione, A., Tedesco, M., Neukom, S., Koudelka-Hep, M., and Martinoia, S. (2009). Active pixel sensor array for high spatio-temporal resolution electrophysiological recordings from single cell to large scale neuronal networks. *Lab on a Chip* 9**,** 2644-2651.

Chiappalone, M., Bove, M., Vato, A., Tedesco, M., and Martinoia, S. (2006). Dissociated cortical networks show spontaneously correlated activity patterns during in vitro development. *Brain Research* 1093**,** 41-53.

Izhikevich, E.M. (2003). Simple model of spiking neurons. *IEEE Transactions on Neural Networks* 6**,** 1569-1572.

Izhikevich, E.M. (2004). Which model to use for cortical spiking neurons? *IEEE Transactions on Neural Networks* 15**,** 1063-1070.

Marom, S., and Shahaf, G. (2002). Development, learning and memory in large random networks of cortical neurons: lessons beyond anatomy. *Quarterly Reviews of Biophysics* 35**,** 63-87.

Pasquale, V., Massobrio, P., Bologna, L.L., Chiappalone, M., and Martinoia, S. (2008). Self-organization and neuronal avalanches in networks of dissociated cortical neurons. *Neuroscience* 153**,** 1354-1369. doi: 10.1016/j.neuroscience.2008.03.050.
