# Supplementary material for: ToolConnect: A Functional Connectivity Toolbox for In vitro Networks
Source: Front Neuroinform. 2016 Mar 30;10:13. doi: 10.3389/fninf.2016.00013 (PMC4811958; doi:10.3389/fninf.2016.00013)
Supplement: Supplementary file 2 [file DataSheet2.DOCX]

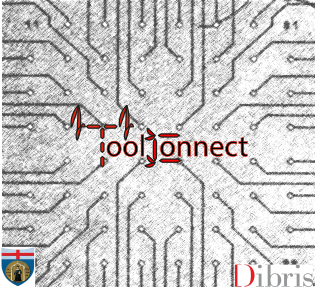
 **ToolConnect**

**User Reference**

**version 1.02**

**13^th^ JANUARY 2016**

**NBT Laboratory, DIBRIS, University of Genova**

**Introduction**

ToolConnect is a software developed in C# to infer functional connectivity of *in-vitro* neural networks coupled to Micro-Electrodes Array (MEAs). The current version of the software (v. 1.01) implements four different connectivity methods to infer the functional connectivity: Cross-Correlation (CC), Partial Correlation (PC), Transfer Entropy (TE) and Joint Entropy (JE). Moreover, ToolConnect includes graphical tools to view and analyze the functional analysis results.

This user guide explains how to use ToolConnect’s main features.

**TABLE OF CONTENTS**

- **Initialization Section**………………………………………………………………............**1**
  - *Minimum platform requirements*………………………………………………........*1*
  - *ToolConnect’s menu description.*……………………………………………........*1*
  - *Multiple Analysis*……..…………………………………………………………......*3*
  - *Input Data directory tree structure*………………………………………………....*3*
  - *Input Data Format*……………………………………………………………….....*4*
  - *Output Data Format*………………………………………………………………..*4*
- **Computational Section**……………..………………………………………………….....**5**
- *Acquisition system recognition and plot*…………………………………………..*5*
  - *Cross/Partial correlation*……………………………………………………….....*6*
  - *Transfer Entropy*……………….……………………………………………….....*9*
  - *Joint Entropy*……………………...…………………………………………….....*10*
- **Graphical Section**……………………………….…………………………………….....**11**
  - *Compute/Thresh Connectivity Matrix*………………………………………….....*11*
  - *Plot Correlogram*………...…………………………………………………….....*15*
  - *Graph Theory Analysis*………………………………………..……………….....*18*
  - *Raster Plot*………………………………………………………….………….....*19*

**Initialization Section**

*Minimum Platform Requirements*

ToolConnect has been implemented as a standalone windows GUI application, using C# programming language with Microsoft Visual Studio based on .NET framework 4.5 development environment. Accordingly, the software requires Microsoft Windows operating system (graphically optimized for versions 8, 8.1 and 10) and Microsoft Visual Studio C# (version 2013 or higher). ToolConnect is an open source software, thus, the downloadable source code is a Visual Studio Project that the user has to import in the workspace. Then, the software can be run with a simple click on the debug button in Visual Studio. There are no minimum hardware requirements to run the software, however, the computational performances are directly related to the hardware platform performances (available RAM and CPU’s clock rate).

*ToolConnect’s menu description*

*ToolConnect’s* GUI offers a drop-down menu that allows the selection and opening of the addressed interfaces designed for the Computational and the Graphical sections (cf. Figure 1 for a menu’s detailed description); these interfaces provide submenus, which make possible to set all the input parameters.

In detail, the main menu’s tabs in Figure 1 are: Open Files, View, Open Windows, Cross/Partial Correlation, Transfer Entropy, Joint Entropy, Compute/Thresh CM and plot Correlograms.

Briefly, Open Files allows the user to choose the folder containing the spike trains files, the View menu includes three different visualization options: Tile Vertically, Tile Horizontally or full window. These visualization options (also including the list of the open interfaces in the menu’s voice Open Windows) allow the simultaneous view of several interfaces; the usefulness of this option arises from the multi-threading implementation strategy of ToolConnect (cf. Sec. Multi Threading Implementation), which makes possible the simultaneous use of both the graphical and the computational section. Cross/Partial Correlation tabs offers the possibility to start the dedicated interfaces for cross and partial correlation analysis in frequency domain (CC/PC in frequency domain) or for the cross-correlation in time domain (CC in Time Domain). Transfer Entropy and Joint Entropy start the interfaces relative to the two information theory based methods, while Compute/Thresh CM and plot Correlograms refer to the graphical Section. The former, has also computational functions, allowing to threshold and plot the CM (menu’s voices Open File -> existent CM) or to compute and plot the CM from the correspondent correlograms (menu’s voices Open File -> Extract CM from correlograms). The latter, gives access to all the options designed for plotting the correlograms

*
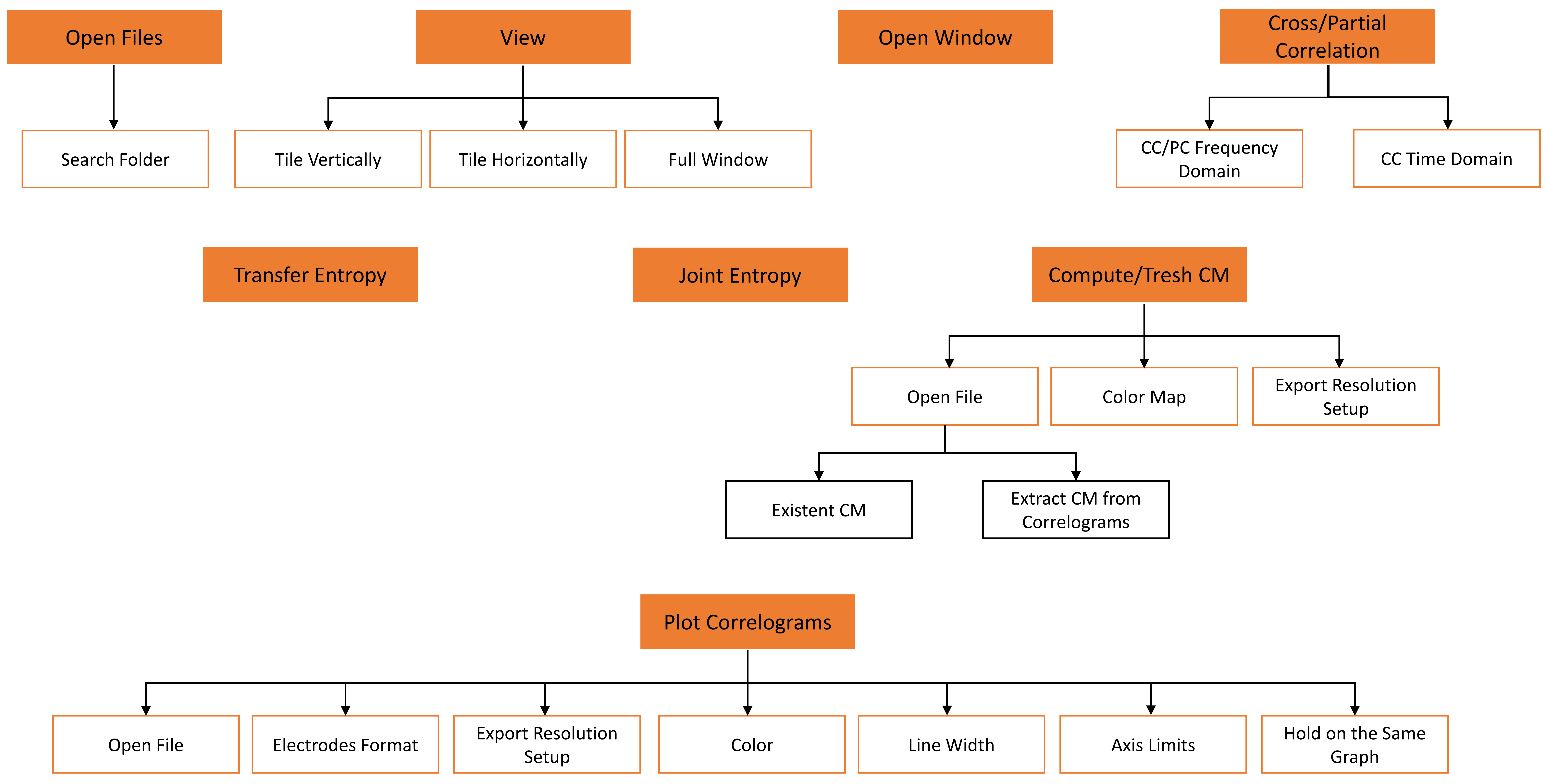
*

Figure 1: TOOLCONNECT’s menu description

*Multiple Analysis*

ToolConnect implements a multiple experimental analysis approach: it consists in an automatic search for the experiments to be analyzed and apply the needed analysis, several times, to different collections of data.

*Input Data directory tree structure*

ToolConnect’s approach to the multiple analysis relies on a specific directory tree structure for the input files (see Figure 2). In detail, a main folder contains all the experiments to analyze (one folder per experiment); in each experiment’s folder there is another folder containing all the phases of the experiment (one folder per phase).


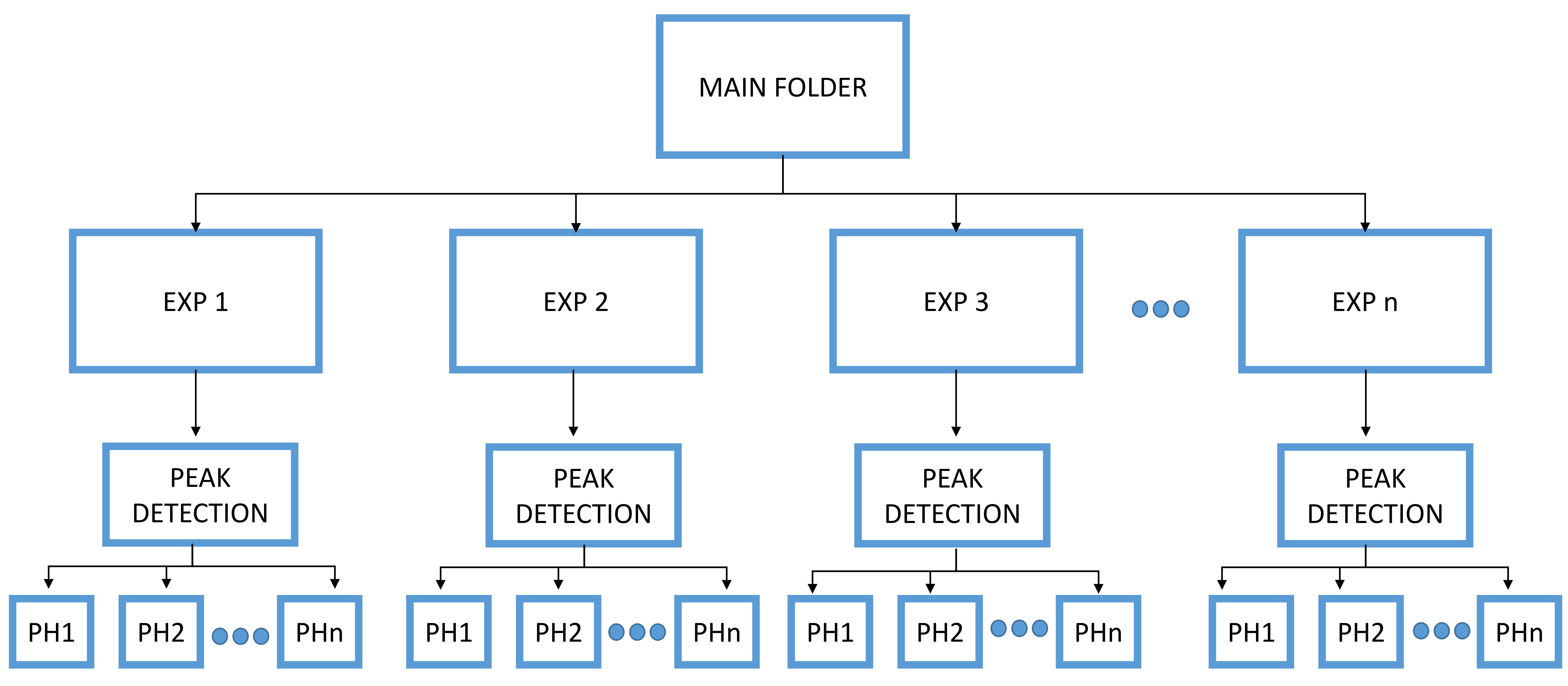


Figure 2. Schematic representation of the input data’s directory tree structure requested by ToolConnect.

*Input Data format*

Spike trains formatted as text files (.txt file format) are the input data of all the computations implemented in ToolConnect. The spike train relative to each channel has to be stored in a single file (i.e., one file per channel). In detail, each file contains a sequence of integers, where the first element is the total number of samples in that experimental session, while the other elements are the time stamps (i.e., the samples correspondent to each recorded spike). Each element of the aforementioned sequence must correspond to a different line of the text file (see Figure 3).


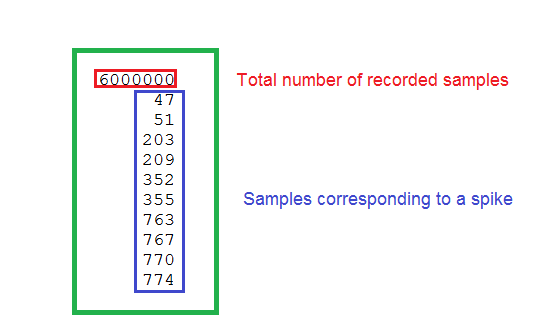


Figure 3. Example of spike train's text file format accepted by ToolConnect.

*Output Data format*

ToolConnect stores the output data preserving the same directory tree structure of the input data (see Figure 4). The name of the parent folder of the directory tree will be the concatenation of the input data main folder‘s name and the date and time relative to the beginning of the analysis. As Figure 4 shows, the output of CC and PC is represented by three connectivity matrices and one folder containing the correlogram of each electrode, formatted as a text file. TE and JE’s output is the connectivity matrix formatted as a text file. All these matrices can be easily imported in MATLAB or in any other software for further analysis.


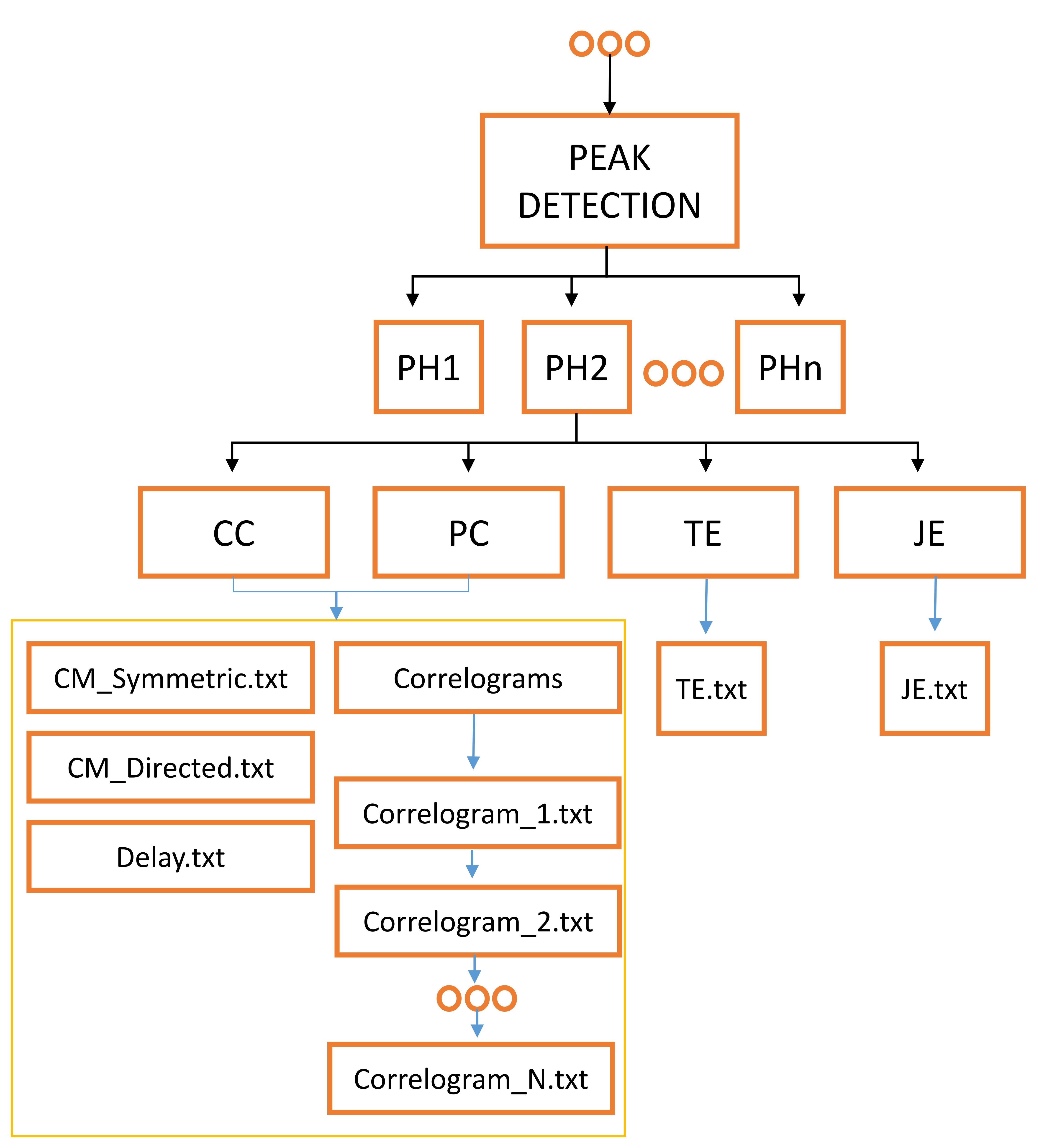


Figure 4. ToolConnect’s ouput directory tree structure.

**Computational Section**

*Acquisition system recognition and plot*

ToolConnect is expected to be independent from the acquisition system used to acquire the electrophysiological data. However, we decided to embed in ToolConnect some widely used acquisition systems, in order to provide the user with the possibility to graphically view and select the electrodes for the analysis (see figure 5). In the current version of ToolConnect, the incorporated acquisition systems are the MEA 60, 120 and 256 channels from Multi-Channel Systems (MCS, Reutlingen, Germany) and the 4096 electrodes recording system from 3brain (Landquart, Switzerland). All the not recognized acquisition systems will not be graphically displayed, but they will be processed automatically considering all the recording electrodes.


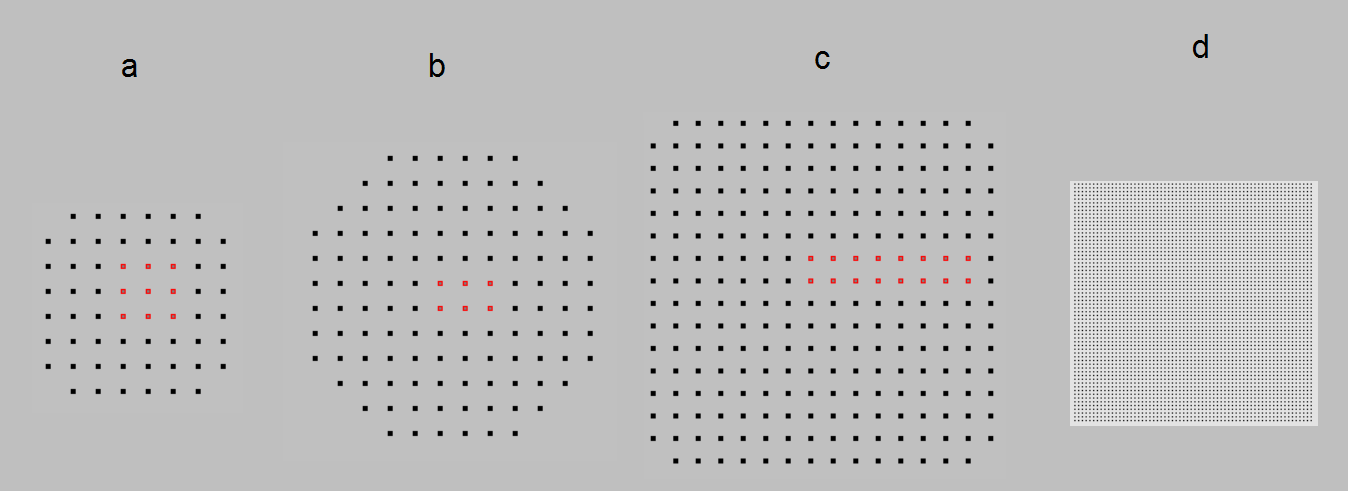


Figure 5. Acquisition system recognized by ToolConnect. Panel a, b, c: MEA 60, MEA 120, MEA 256 Multi-Channel Systems. Panel d: 3Brain’s APS recording system.

*Cross/Partial correlation*

To perform a cross or partial correlation analysis the user must select the main folder of the directory tree previously described, using "Open files" from the menu.

If the acquisition system is recognized (see *Acquisition system recognition and plot),* at least two electrodes must be selected in the interface in the bottom left. Then, open the dropdown menu with a right click in the interface and click “Selection completed”. You can also select or deselect all the electrodes through the correspondent voices in the aforementioned menu. If you want to change the selection, use the right click to reopen the drop down menu and select the voice new selection (every running connectivity algorithm will be aborted).


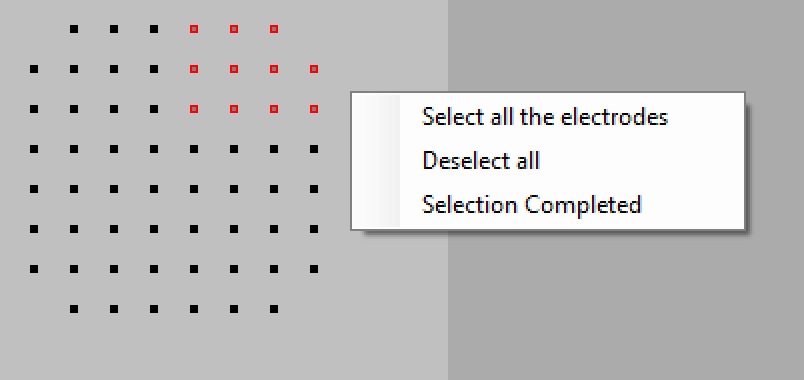


If the acquisition system is not recognized (but directory tree is recognized) the software alerts the user throwing a warning (see image below) and allows the analysis of all the recorded electrodes.


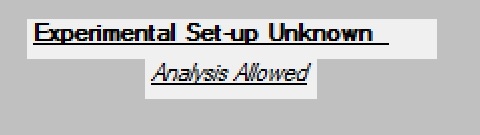


- To compute a Cross/Partial correlation in Frequency domain:

1. In ToolConnect, click the "Cross/Partial Correlation" tab in the top bar then select "CC/PC Frequency Domain".


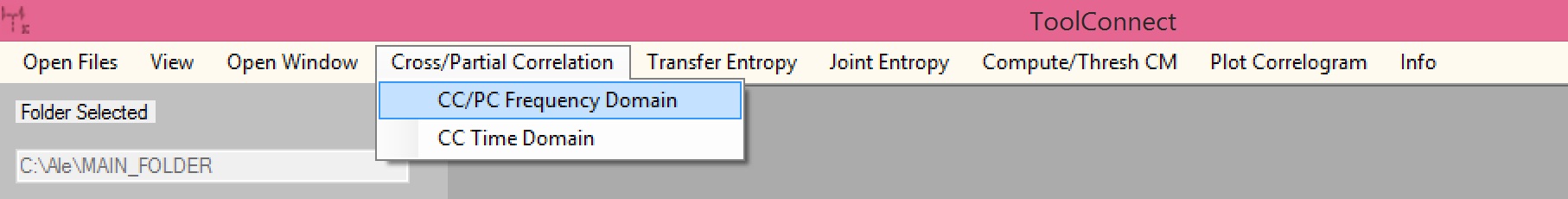


2. The analysis requires several parameters: bin size, sampling frequency, correlation window’s size and the overlap percentage (refers to the paper for much details). Insert the values appropriately.


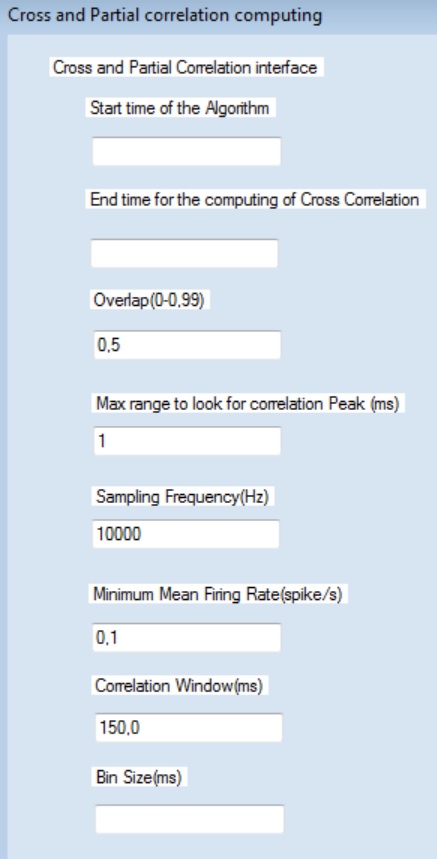


3. Click the Compute button (This process may take some time depending on the values chosen for the parameters, the firing rate of the recorded spike trains and the algorithm selected to process the data.

- To compute a Cross/Partial Correlation in Time domain:

1. In ToolConnect, click the "Cross/Partial Correlation" tab in the top bar then "CC Time Domain".


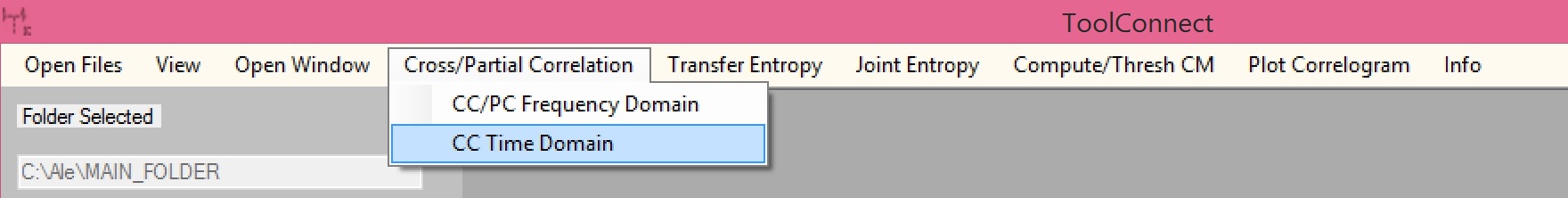


2. Enter values appropriately.

3. Insert the bin size used for the computation in the "Bin Size" textbox.


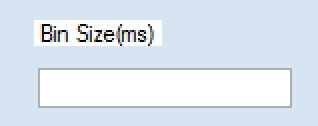


4. Click the "Compute" button (This process may take some time depending on bin size chosen and the PC’s hardware).

*Transfer Entropy*

In order to compute a Transfer Entropy a data folder must be chosen using "Open files" from the top bar and then at least two electrodes must be selected in the interface in the bottom left (if the acquisition system is recognized, see section *Cross- and Partial Correlation*).


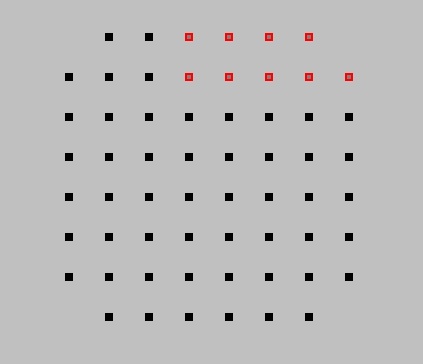


To compute a transfer entropy:

1. In ToolConnect, click the "Transfer Entropy" tab in the top bar.


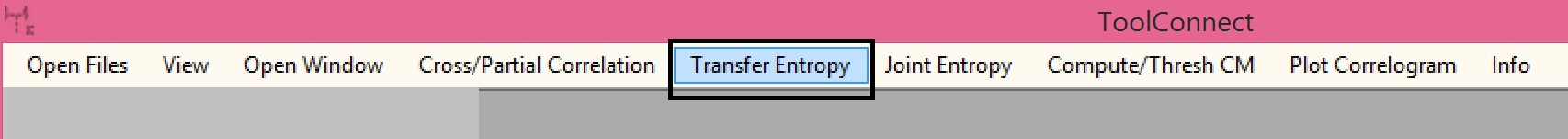


2. Enter values appropriately.

3. Insert the bin size used for the computation in the "Bin Size" textbox.


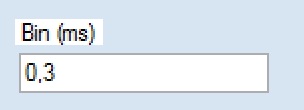


4. Click the "Compute" button (This process may take some time depending on bin size chosen and pc’s hardware).

*Joint Entropy*

In order to compute a Joint Entropy a data folder must be chosen using "Open files" from the top bar and then at least two electrodes must be selected in the interface in the bottom left (if the acquisition system is recognized, see section *Cross- and Partial Correlation*).


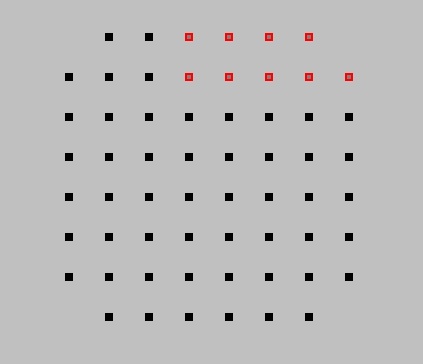


To compute a joint entropy:

1. In ToolConnect, click the "joint entropy" tab in the top bar.


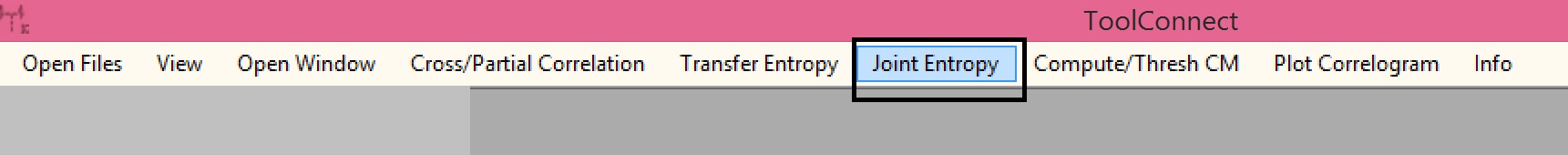


2. Enter values appropriately.

3. Insert the bin size used for the computation in the "Bin Size" textbox.


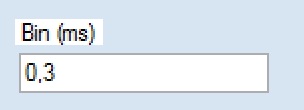


4. Click the "Compute" button (This process may take some time depending on bin size chosen and pc performances).

**Graphical Section**

*Compute/Thresh Connectivity Matrix*

ToolConnect includes graphical tools for the Connectivity Matrices analysis (see figure 6). In particular, if the user has the Connectivity Matrix’s text file (output of all ToolConnect’s embedded connectivity methods) he can apply a threshold and graphically view the CM, the thresholded CM and the correspondent graph.

To threshold and graphically view the connectivity matrix and graph:

1. In ToolConnect, click the "Compute/Thresh CM" tab in the menu.


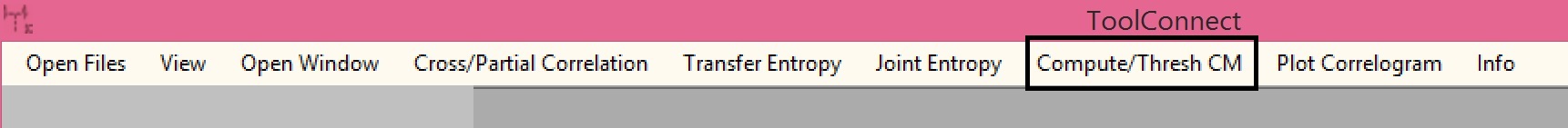


2. Click the "Options" tab, then the "Open file" then the "Existent CM" and select the text file corresponding to the CM to analyze.


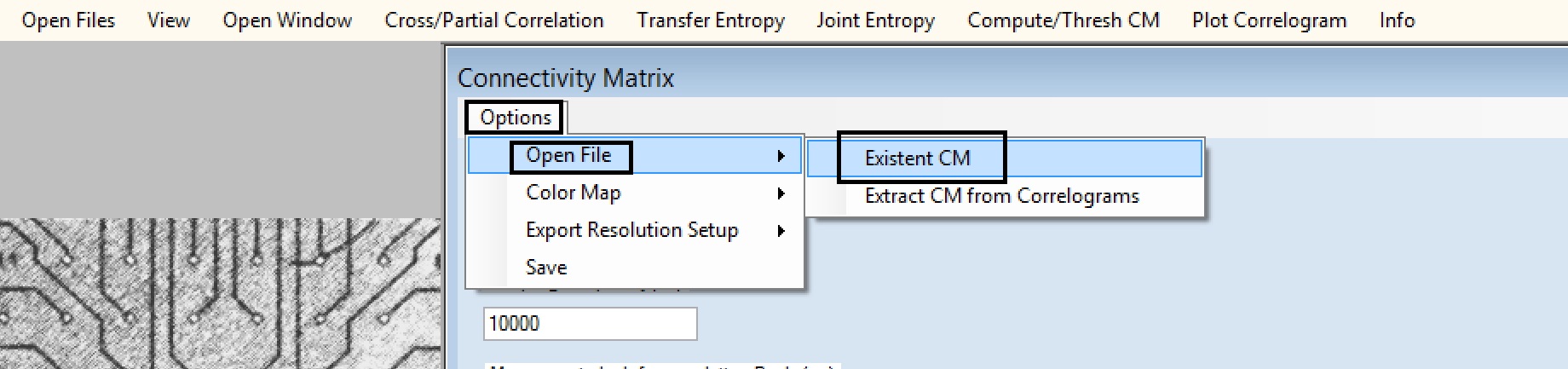


3. Select the "Multi Channel System" check box if the acquisition system used for the recordings correspondent to the analyzed CM comes from Multi Channel Systems (necessary for the specific electrodes’ numeration).


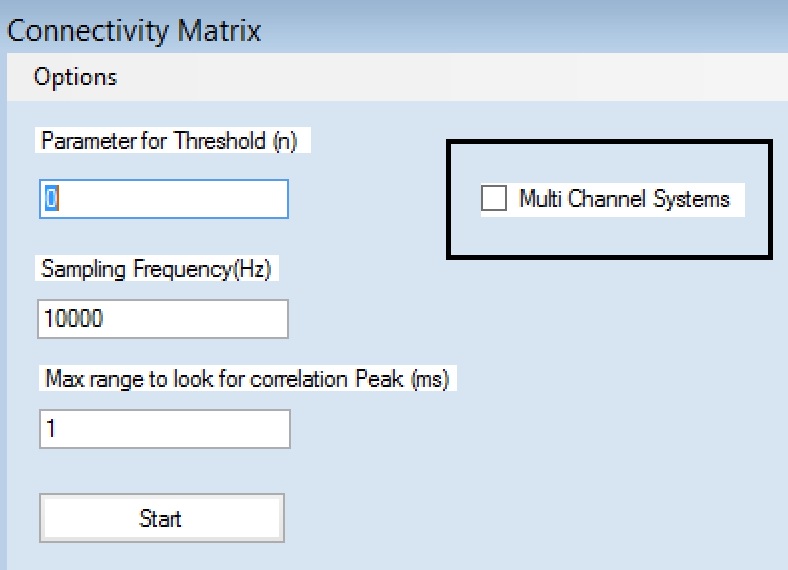


4. Click the "Start" button.


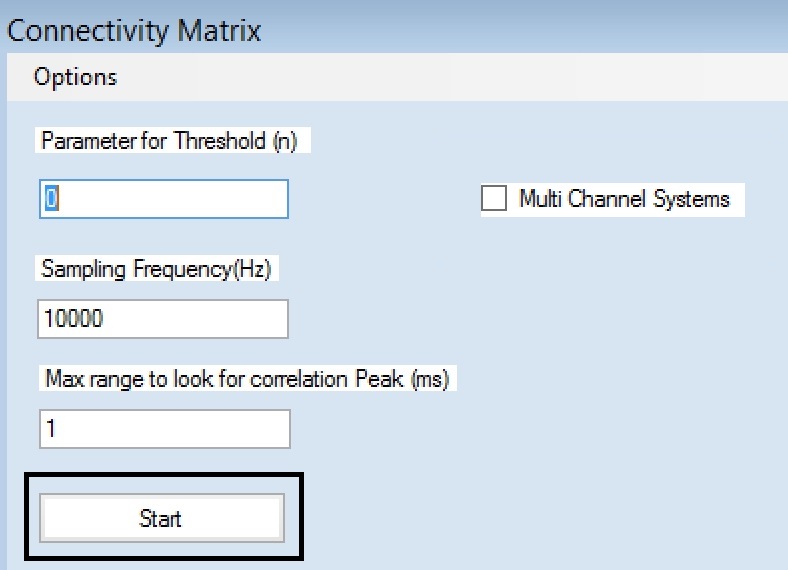


ToolConnect offers also the possibility to compute, threshold and graphically view a CM, directly from the correlograms coming from a cross- or partial correlation analysis.

To compute directly a CM from the correlograms:

1. In ToolConnect, click the "Compute/Thresh CM" tab in the top bar.


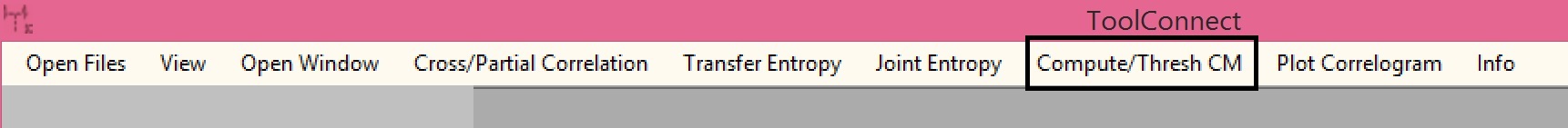


2. Click "Options" tab, "Open file" and then "Extract CM from correlograms". At this stage, select the folder containing the cross or partial correlograms’ text files, produced as output for cross-and partial correlation analysis.


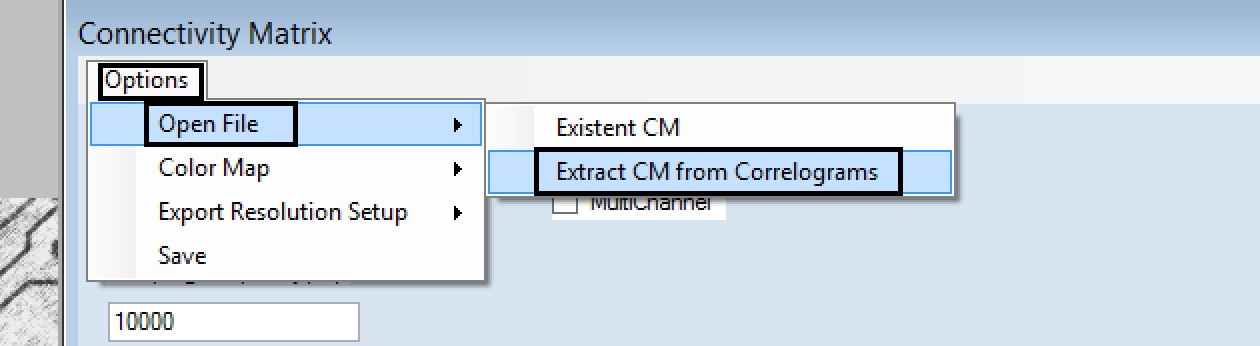


3. Select the "Multi Channel System" check box if the acquisition system used for the recordings correspondent to the analyzed CM comes from Multi Channel Systems (necessary for the specific electrodes’ numeration).


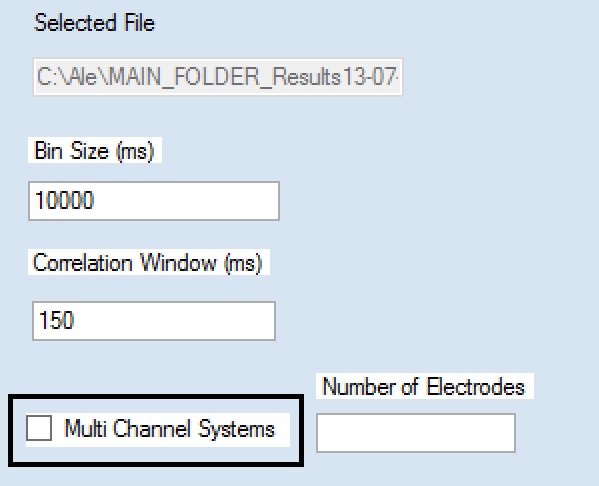


4. Insert the number of electrodes used in the cross- and partial correlation analysis in the "Number of Electrodes" textbox (otherwise, an error will arise).


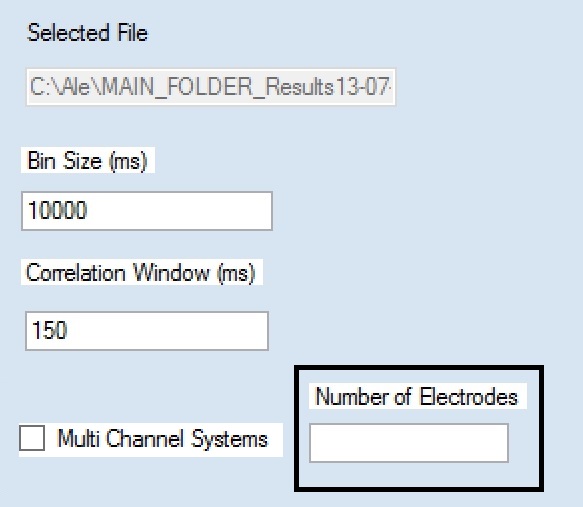


5. Click the "Start" button.


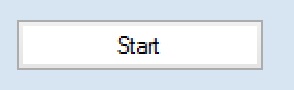


"Options" tab gives the user with the possibility to personalize the color map used to plot the matrix in false colors (i.e., with colors proportional to the values assumed by its elements) choose the resolution for exporting the CM’s and Connectivity Graph’s images.


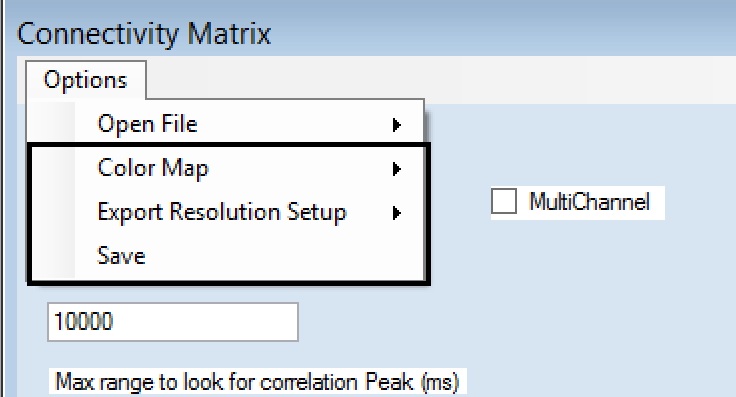


a


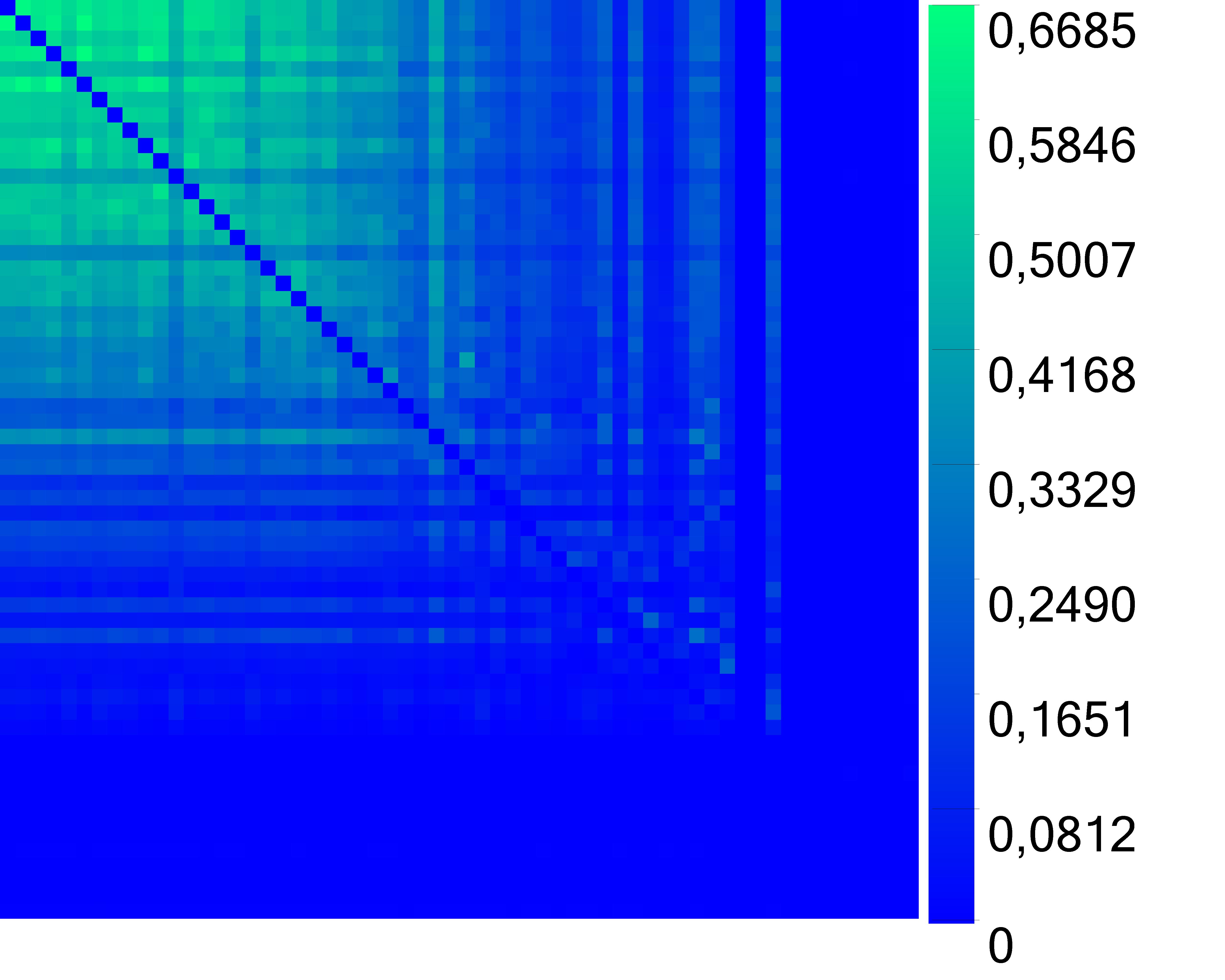

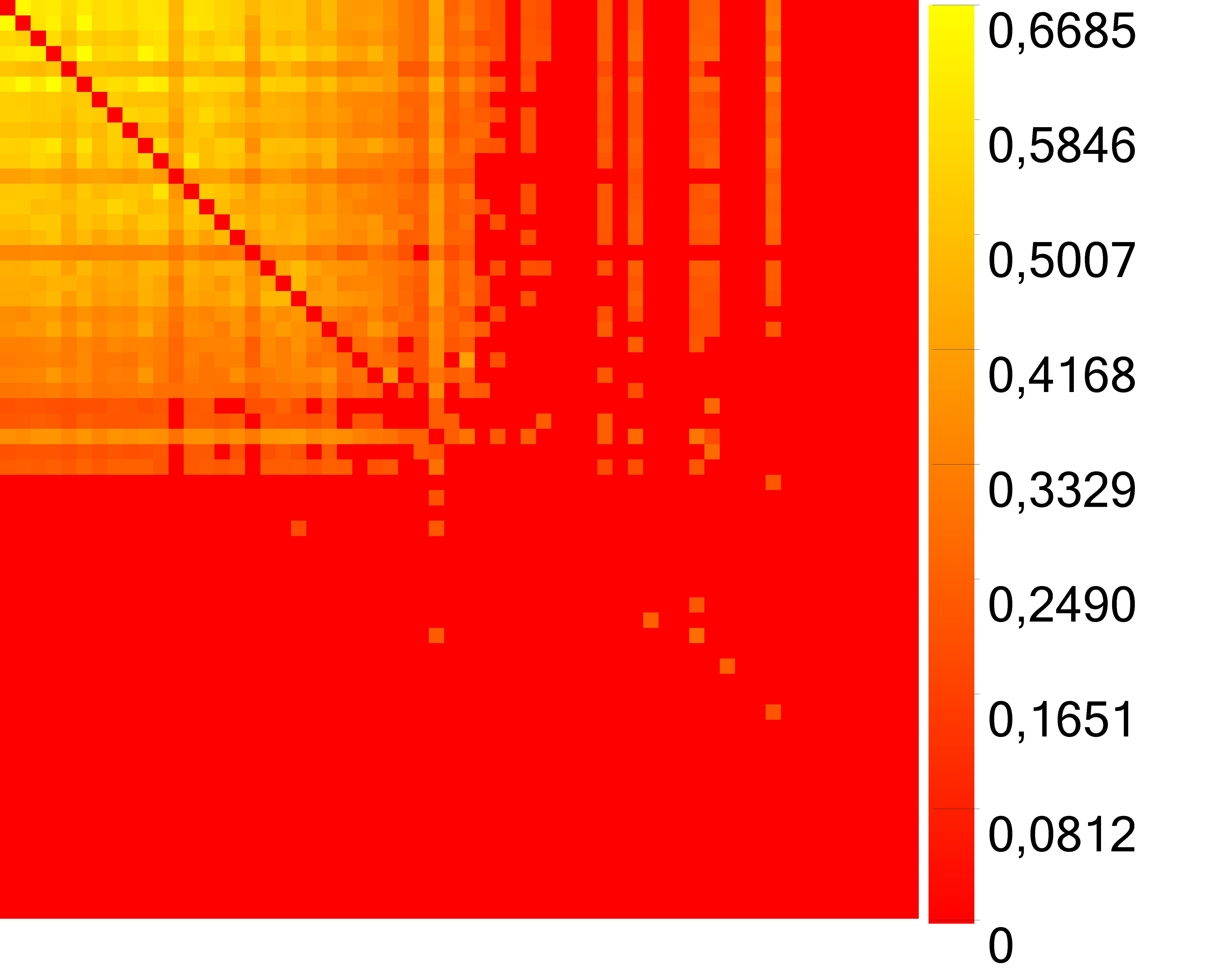

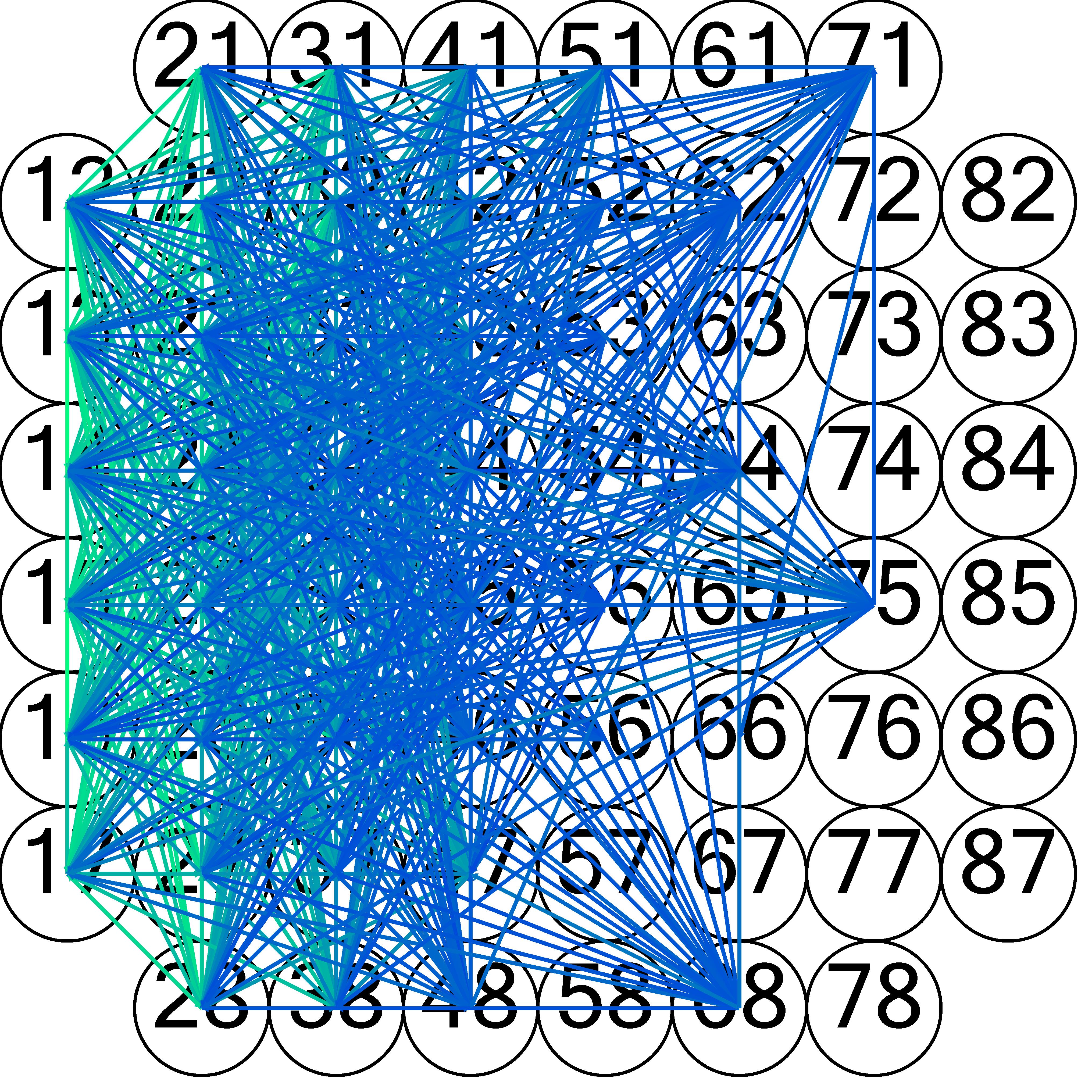


b

c

Figure 6. Example of ToolConnect’s connectivity matrix. Panel a, CM. Panel b, TCM, Panel c, Connectivity graph.

*Plot Correlogram*

As previously stated, cross- and partial correlation analysis produce one correlogram file for each analyzed electrode. In detail, each electrode’s correlogram is a text file containing the correlograms obtained by computing the cross- or partial correlation for the electrode at issue versus all the other recording electrodes. In this text file, each correlogram is preceded by the numerical index of the electrode considered as post-synaptic in the analysis.

ToolConnect incorporates a graphical tool for plotting the correlograms (see figure 7). To plot a Correlogram:

1. In ToolConnect, click the "Plot correlogram" tab in the menu.


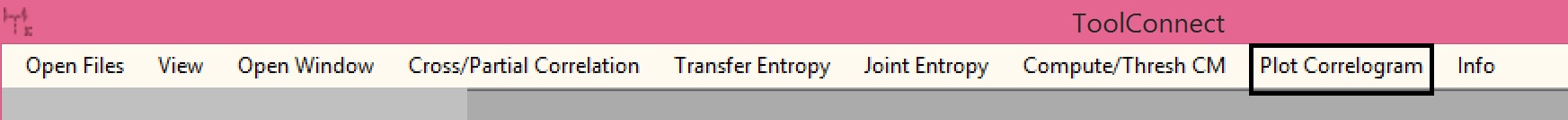


1. Click the "Open folder" tab, then the "Search" and select a folder containing cross or partial correlograms (if the folder doesn't contain a correlogram an error window will appear).


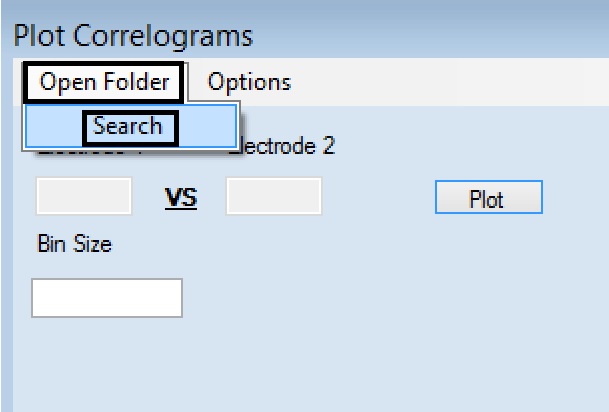


3. Choose two electrodes by clicking on the matrix or insert them manually in the provided text boxes. Silent electrodes are shown in gray. Silent electrodes are those that have a mean firing rate lower than the minimum mean firing rate indicated in the analysis’s interfaces.


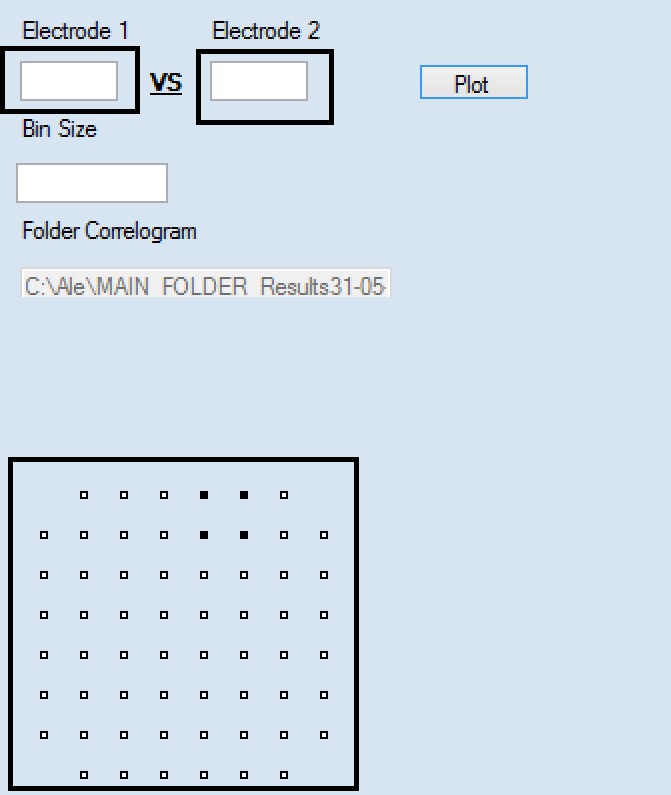


4. Insert the bin size used for the cross- or partial correlation analysis in the "Bin Size" textbox.


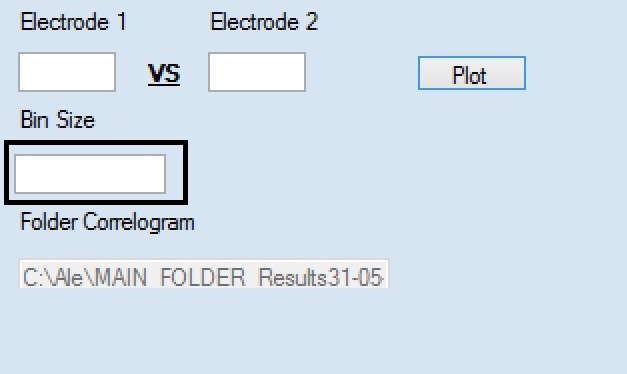


5. Click the "Plot" button (if the electrodes selected are not contained in the correlogram an error window will appear).


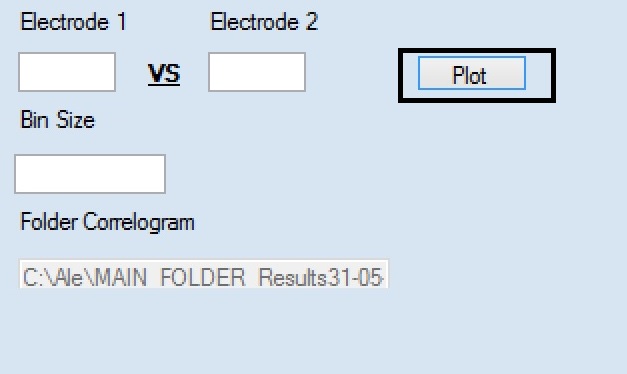


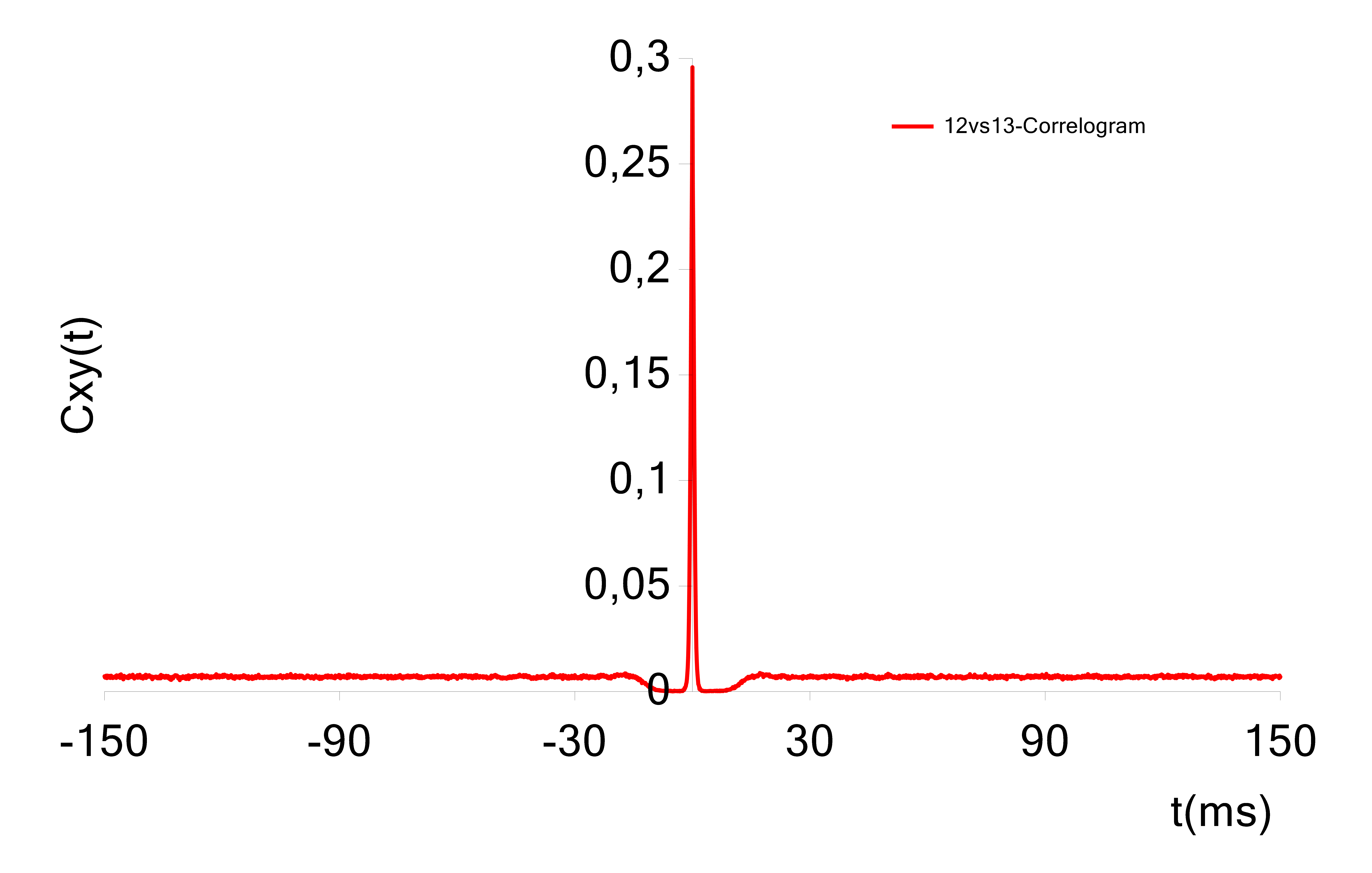


Figure 7. Example of Cross-correlogram produced by ToolConnect.

"Options" tab provide the user with the possibility to personalize the plot. The user can choose the color, the line width and the axis limits, as well as the resolution for saving the correlogram image.


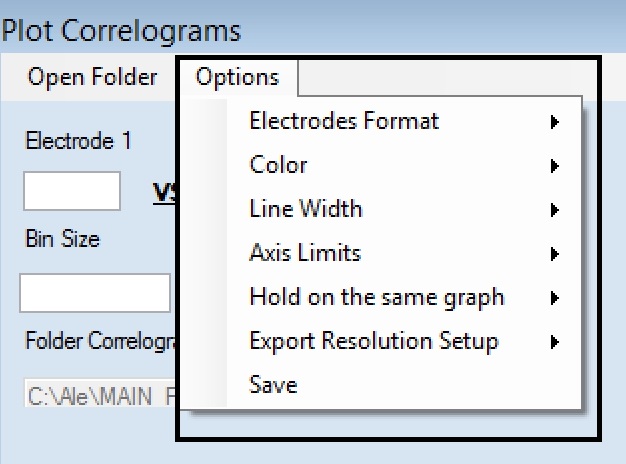


*Graph Theory Analysis*

ToolConnect provides the user with the possibility to perform graph theory’s analysis. In particular, it is possible to apply the more common graph theory’s metrics to the Thresholded Connectivity Matrix (TCM), described before. In the current version (v. 1.0.1), ToolConnect supports the computation of the Cluster Coefficient (CC), the Path Length (PL), the number of links and nodes detected by the connectivity methods and the degree distribution (i.e., the number of in-going and out-going links for each analyzed electrode). The graph theory analysis’s output is a single value for the CC, the number of links and neurons; the mean value and the *n* x *n* matrix (where *n* is the total number of electrodes) of the values relative to each possible pair of electrodes for the PL. Finally, the analysis produces a text file reporting the in-degree, the out-degree and the total degree for each electrode (one row per electrode).

1. In ToolConnect, click the “Graph Theory Analysis" tab in the menu.


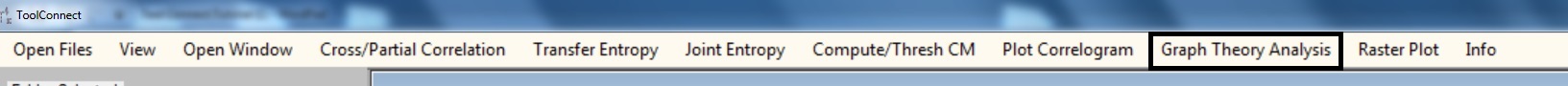


1. Click the "Open" tab, then the "TCM" and select a folder containing the Thresholded Connectivity Matrix.


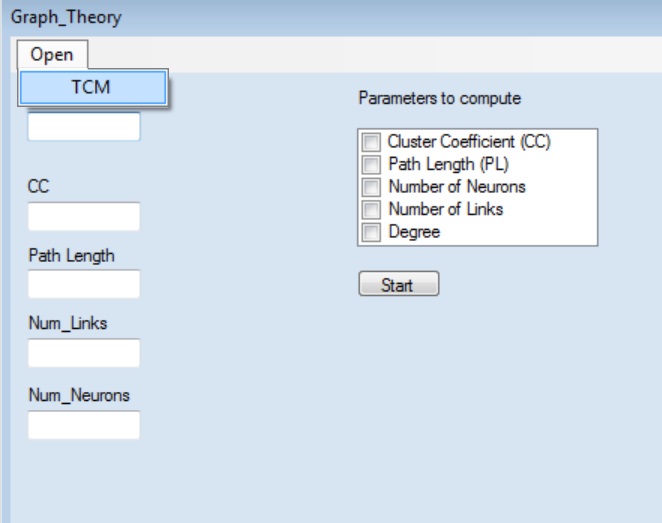


1. Select parameters to compute and the click “Start” button.


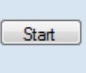


*Raster Plot*

ToolConnect implements the possibility to compute and represent the raster plot (see figure 8).

1. In ToolConnect , click the “Raster plot" tab in the menu.


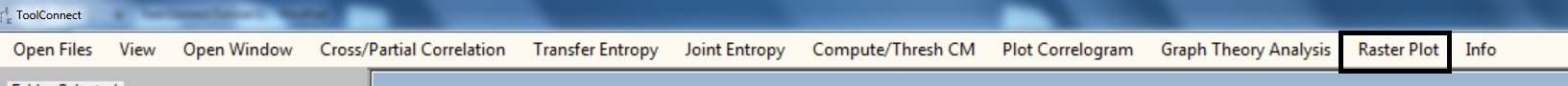


1. Click the "Open" tab, then select the folder containing the peak trains to analyze.

**
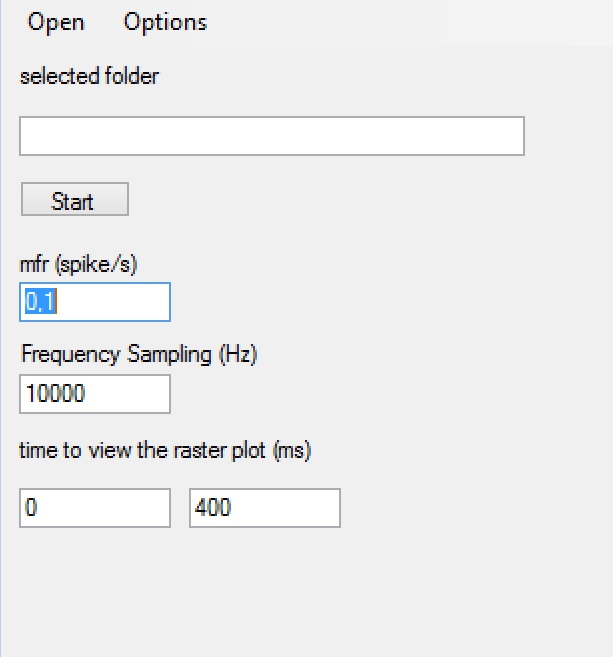
**

1. Insert the minimum mean firing rate (mfr) to consider that an electrode is active. Only the electrodes with an mfr higher than the value indicated will be considered for the raster plot.
2. Insert the sampling frequency and the raster plot’s time interval.
3. Click Start button.

**
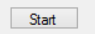
**


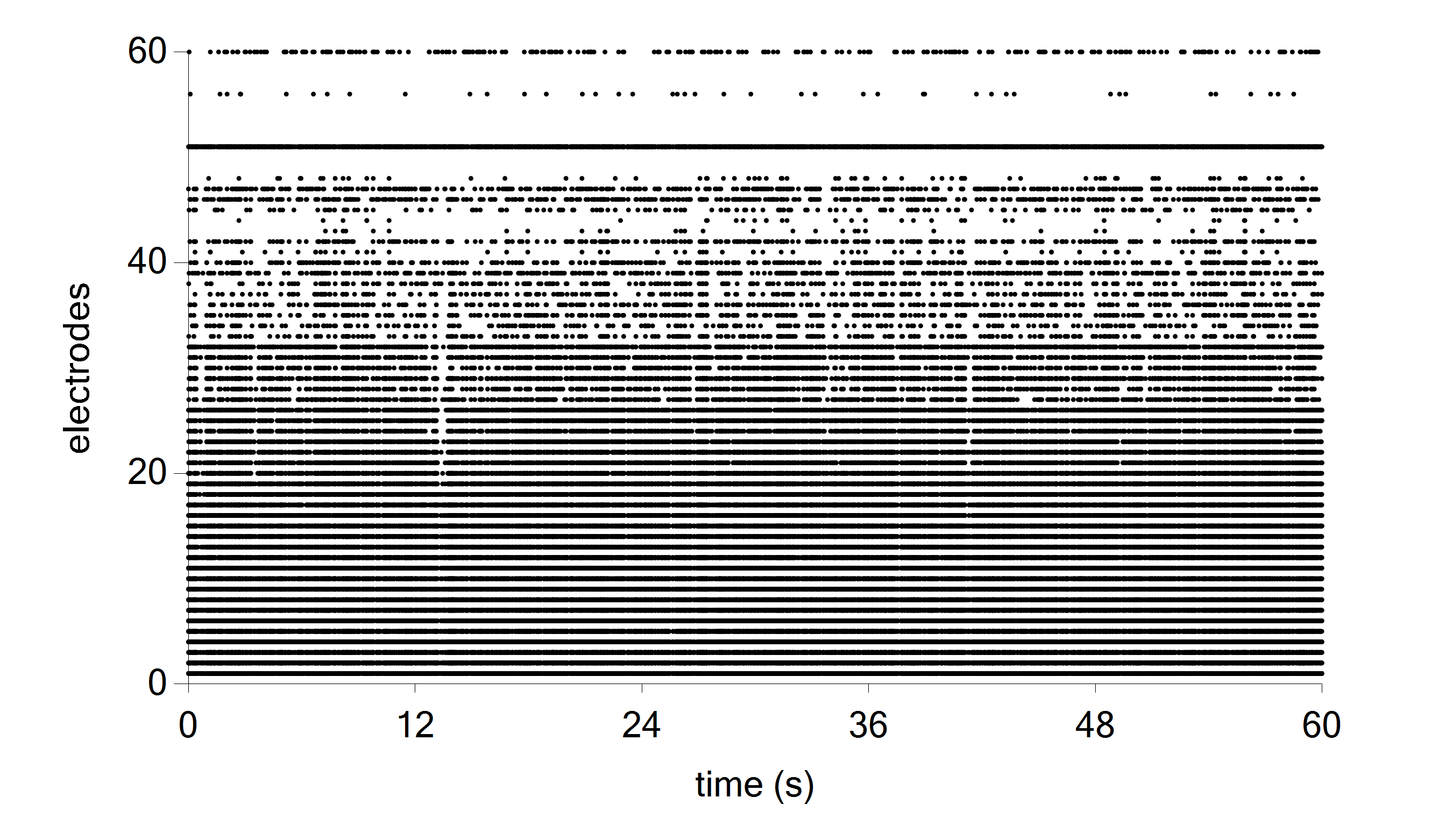


Figure 8. Example of ToolConnect’s raster plot.

**CONTACT INFORMATION**

**Department of Informatics, Bioengineering, Robotics and System Engineering (DIBRIS)**

University of Genova

[**www.dibris.unige.it**](http://www.dibris.unige.it)

**ToolConnect ’s Authors**

Vito Paolo Pastore, Daniele Poli, Aleksandar Godjoski, Sergio Martinoia and Paolo Massobrio

**Contacts**

[**vito.paolo.pastore@edu.unige.it**](mailto:vito.paolo.pastore@edu.unige.it)

[**paolo.massobrio@unige.it**](mailto:paolo.massobrio@unige.it)

**User Guide Author**

Vito Paolo Pastore
